# Age-specific exposure to human-induced increases in humid heat

**DOI:** 10.1126/sciadv.aeb3232

**Published:** 2026-08-26

**Authors:** Rosa Pietroiusti, Erich Fischer, Rupert Stuart-Smith, Luke J. Harrington, Chloe Brimicombe, Matthias Mengel, Annalisa Savaresi, Sam Adelman, Wim Thiery

**Affiliations:** ^1^ Department of Water and Climate, Vrije Universiteit Brussel, Brussels, Belgium.; ^2^ Institute for Atmospheric and Climate Science, ETH Zurich, Zurich, Switzerland.; ^3^ Oxford Sustainable Law Programme, University of Oxford, Oxford, UK.; ^4^ Te Aka Mātuatua School of Science, University of Waikato, Hamilton, New Zealand.; ^5^ Department of Social Policy Intervention, University of Oxford, Oxford, UK.; ^6^ Potsdam Institute for Climate Impact Research, Potsdam, Germany.; ^7^ Centre for Climate Change, Environmental and Energy Law, University of Eastern Finland, Joensuu, Finland.; ^8^ Faculty of Arts and Humanities, University of Stirling, Stirling, UK.; ^9^ School of Law, University of Warwick, Coventry, UK.

## Abstract

Children are particularly vulnerable to climate change impacts and will face intensifying effects of climate change during their lifetimes. Here, we show that children already face disproportionate exposure compared to older generations, as many live in regions hit the hardest by escalating humid heat stress. Combining demographic data with a global grid-scale attribution framework, we find that, at present-day warming levels, 2.8 to 3 times more children aged 0 to 9 years than adults aged 60 to 69 years are exposed to at least 20 additional heat stress days attributable to anthropogenic climate change based on both reanalysis (3.0 [3.0 to 3.1]) and climate models (2.8 [2.4 to 3.0]). On average, children experience 9 to 10 more attributable heat stress days than people aged 60 to 69 years (reanalysis: 9 [6 to 9]; models: 10 [6 to 13]), and this difference is likely conservative as children spend more time outdoors. This intergenerational disparity persists as overall heat stress escalates under global warming of 1.5° and 2°C, highlighting the need for mitigation and adaptation policies to protect children from heat-related health risks.

## INTRODUCTION

Climate change is increasing the frequency, intensity, and duration of dry and humid heat extremes worldwide (*1*). For instance, the 2021 Pacific Northwest heatwave and the humid heatwave of 2023 in South and Southeast Asia, which each caused hundreds of fatalities (*2*, *3*), were both made more likely and intense by anthropogenic climate change (*4*, *5*). The heat-related health burden of climate change is accordingly becoming increasingly evident. For example, anthropogenic climate change is already driving increased heat-related mortality (*6*, *7*), which is projected to be the greatest climate change–related burden of death in many parts of the world (*8*).

In a heat-health context, heat stress refers to environmental conditions (e.g., temperature, humidity, solar radiation, and wind) and personal factors (e.g., activity and clothing) that impose a net thermal load on the body (see Box 1) (*9*). Exposure to heat stress can lead to a range of adverse health outcomes in humans, particularly on vulnerable groups and populations facing structural barriers to heat adaptation. Excessive heat challenges the body’s ability to thermoregulate, aggravates preexisting cardiovascular and respiratory diseases, and leads to increased risk of heat stroke, kidney failure, and lung damage (*10*). When high temperatures occur in combination with high humidity, evaporative cooling via sweating becomes less effective, increasing the risk of dangerous heat accumulation (*9*–*11*).

Box 1. Key heat-related terminology in atmospheric sciences and human biometeorology.

**Heat**: In human biometeorology, heat refers to energy exchanged between the human body and its surrounding environment or generated internally through metabolism (*9*). Heat transfer occurs via conduction, convection, radiation, and evaporation (sweating) and is influenced by air temperature, humidity, radiation (including solar radiation), and wind speed (*9**,* *11**,* *14*). Together with metabolic heat production, these fluxes determine the human heat balance. When heat gains exceed losses, body temperature can rise (*9*).

**Humid heat**: It describes conditions of high air temperature combined with high humidity. Elevated humidity reduces evaporative heat loss via sweating, the primary method of human heat dissipation (∼75%) (*11*). Low humidity facilitates evaporative cooling but may increase dehydration risk (*9*).

**Heat stress**: It refers to the net heat load imposed on the body when heat gains exceed its capacity for heat dissipation, which can lead to negative effects on an individual (*9**,* *44*). Various heat stress indices exist to represent the combined effect of ambient conditions on the human heat balance.

**Heat strain:** It describes the physiological responses to heat stress (*11**,* *44*), such as increased core and skin temperature, heart rate, and sweat rate. Heat strain can progress to heat illness, such as heat cramps, heat exhaustion, or heat stroke (*9*).

Vulnerability to heat stress varies with age. Exposure to high temperatures is associated with increased morbidity and mortality, particularly among older adults (≥65 years old) and neonates, and with increased morbidity in young children (aged 0 to 4 years) (*7*, *10*, *12*–*17*).

Children are particularly vulnerable to a range of heat-related impacts, with potential lifelong consequences resulting from being in developmental stages. At extreme high temperatures and in hot and humid conditions, young children (0 to 4 years old) can be at higher risk of heat illness (*13*, *18*) because of a reduced sweating capacity and a higher surface area–to–body mass ratio (*13*). Behavioral factors, such as insufficient hydration, more time spent outdoors, and dependence on adult care (*10*, *17*, *19*, *20*), further heighten heat risks for children. Excessive heat has been shown to aggravate asthma, respiratory illness, and kidney-related diseases in children (*16*, *17*, *19*–*21*); to negatively affect learning abilities (*16*, *19*, *22*); and to have negative repercussions on mental health (*18*, *19*). Exposure to excess heat during pregnancy poses risks for birth outcomes, including preterm birth, low birth weight, and stillbirth (*16*, *23*, *24*), highlighting that exposure to heat stress can have lifelong consequences even before birth.

Indirect effects of heat are also important, particularly in low- and middle-income countries (LMICs), where high temperatures can exacerbate water and food insecurity, compromise child nutrition (*25*, *26*), and increase the risk of diarrheal and vector-borne diseases (*8*, *19*, *27*–*30*). These effects have long-term consequences for growth and development (*19*, *26*) and contribute to key risk factors for child mortality (*29*, *31*). Children in low-income settings are particularly vulnerable to heat because of preexisting burdens of infection, undernutrition, limited health care access, and inadequate housing and adaptation measures to heat (*8*, *32*). In high-income countries, heat-related mortality is typically concentrated in older adults, who generally exhibit the highest in relative risk from heat-related mortality worldwide (*7*, *10*). In LMICs, high temperatures can increase mortality in children and young adults through both direct and indirect pathways, representing a considerable health burden, particularly when assessed in terms of total deaths or years of life lost, and in light of younger age structures of the population (*32*–*34*). The evidence base on heat-related health impacts, particularly among children, remains concentrated in high-income countries, with less research in LMICs (*21*).

Global-scale attribution studies have previously analyzed trends and frequency changes of dry-bulb temperature (*35*–*39*) and humid heat extremes (*40*). Yet, how the exposure of people to attributable changes in heat stress varies across age groups has not been assessed. Here, we quantify the age-specific exposure to heat stress attributable to anthropogenic climate change under present-day warming levels, as well as at 1.5° and 2°C of global warming, by integrating demographic data with a global grid-scale attribution framework. To this end, we use a heat stress metric that accounts for temperature and humidity, namely a modified version of the wet bulb globe temperature (WBGT) that assumes moderate winds and shaded conditions (the so-called “indoor” formulation). We find that the world’s youngest people are globally disproportionately exposed to attributable heat stress and that this intergenerational difference persists under near-term warming of 1.5° and 2°C. This provides further evidence on the globally unequal consequences of anthropogenic climate change, revealing a global age-based asymmetry between responsibility for climate change and exposure to its effects.

## RESULTS

### Highest exposure of children at low latitudes

**Fig. 1.**
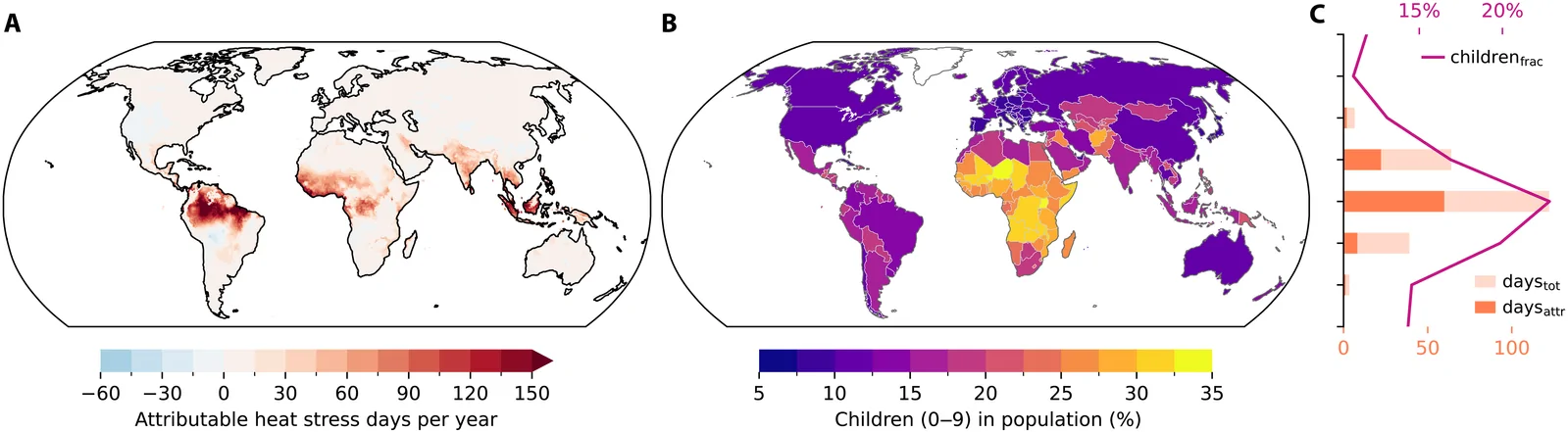
Lower-latitude countries have the youngest populations and the greatest exposure to absolute and attributable humid heat stress. (**A**) Number of attributable heat stress days per year, defined as WBGT > 28°C, at present-day warming levels, estimated from the reanalysis product 20CRv3-W5E5. (**B**) Percentage of children aged 0 to 9 years in national population in 2023. (**C**) Zonal average number of total heat stress days (days_tot_) and attributable heat stress days (days_attr_) per year, defined as WBGT > 28°C, under present-day (2023) warming levels and percentage of children aged 0 to 9 years in population (children_frac_) for 20° latitude bands from 70°S to 50°S to 70°N to 90°N.

We find that the greatest increase in the frequency of heat stress days resulting from anthropogenic climate change has occurred in low-latitude land areas (Fig. 1A and figs. S1 and S2). This is in line with previous research that has consistently found faster increases and earlier emergence of hot extremes at lower latitudes [e.g., (*37**,* *39**,* *41**,* *42*)]. To identify heat stress days, we select days with maximum WBGT exceeding the International Organization for Standardization (ISO) standard threshold for moderate heat stress (WBGT > 28°C) (*43*, *44*), representing conditions under which protective adaptations such as reduced activity or structured rest periods are recommended to prevent heat-related illness. We later test the sensitivity of our results to alternative thresholds.

We quantify attributable heat stress days from three reanalysis products using a statistical method inspired by extreme event attribution. In particular, we model nonstationarity in WBGT as a function of global mean surface temperature (GMST), independently for each calendar month and each grid cell. We then define distributions of WBGT at present-day and preindustrial GMST and estimate the expected frequency of exceedance of the heat stress threshold at present-day warming and in a counterfactual preindustrial climate. The difference in frequency thus represents the fraction of expected occurrences attributable to anthropogenic climate change. We estimate attributable changes separately for each reanalysis product and quantify uncertainty arising from differences between datasets (see Materials and Methods and note S1).

Low-latitude countries are experiencing substantially more heat stress days. For example, in Côte d’Ivoire in West Africa, people are estimated to experience 112 heat stress days per year ([68 to 121], median and range across reanalyses) under present-day warming, compared to 51 days ([15 to 65]) under preindustrial conditions, implying 53 days per year ([47 to 70]) are attributable to human-induced climate change. In contrast, in Germany, people are estimated to experience only 0.1 ([0.1 to 0.2]) heat stress days per year, largely attributable to anthropogenic climate change. This contrast is relevant for age-specific exposure patterns as low-latitude countries have younger populations (Fig. 1B). In Côte d’Ivoire, 27% of the population is aged 0 to 9 years, while in Germany, this age group represents only 9% of the population.

Moving from the country-level to zonal (latitudinal) averages, we find that lower-latitude land areas today experience the greatest total exposure, as well as the greatest attributable exposure to heat stress (Fig. 1C and fig. S3). Lower-latitude land areas have a higher signal-to-noise ratio because of less seasonal variability, leading to higher attributable changes in frequency (*36*, *39*). These areas also have higher baseline levels of heat stress and more frequent crossing of impact-relevant heat stress thresholds, leading to higher absolute and attributable levels of heat stress.

**Fig. 2.**
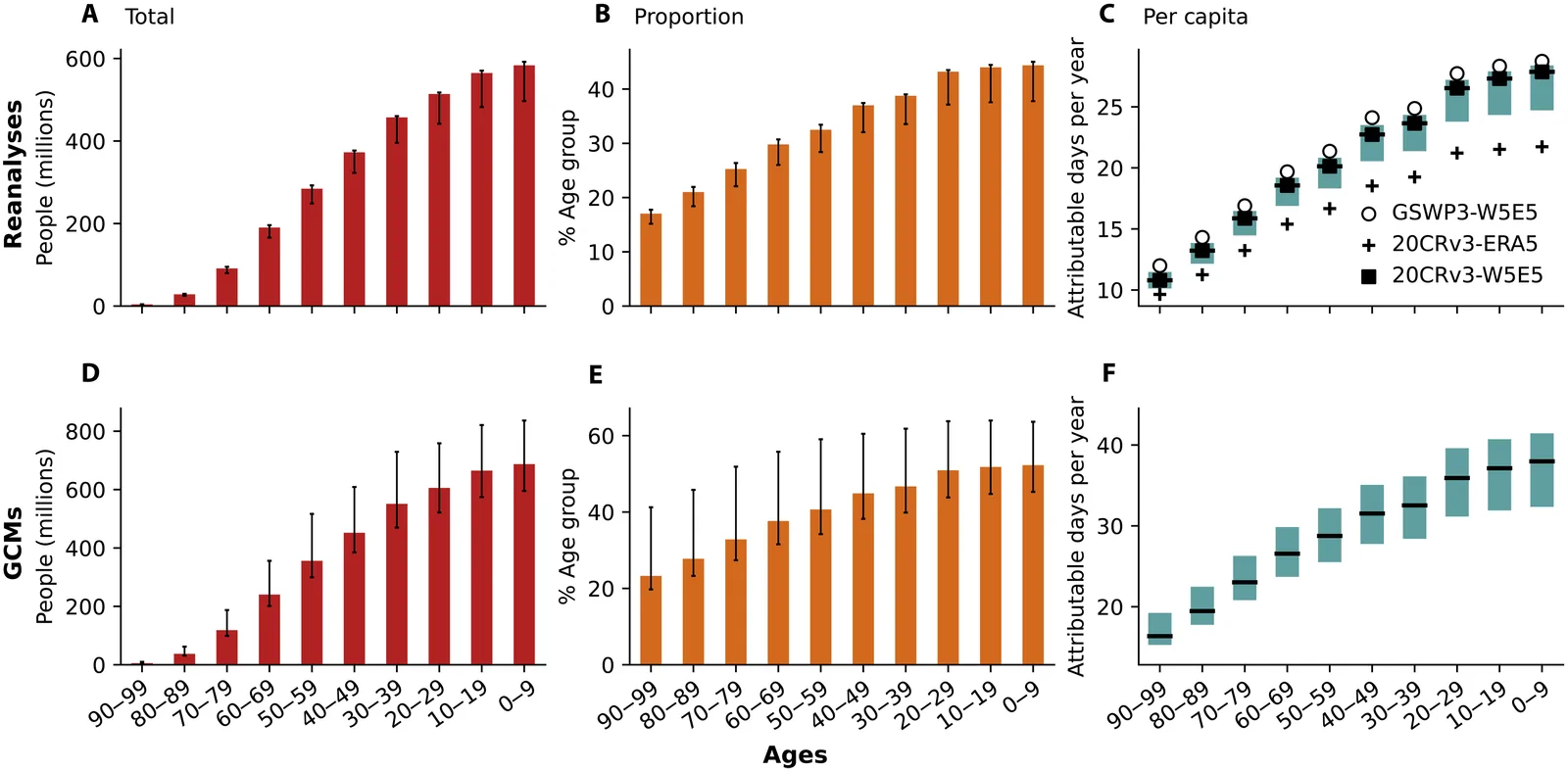
Younger age groups are disproportionately exposed to attributable heat stress. (**A** and **D**) Total number of people of each age group exposed to at least 20 attributable heat stress days per year, defined as WBGT > 28°C. (**B** and **E**) Proportion of the age group exposed to at least 20 attributable heat stress days (WBGT > 28°C) per year. (**C** and **F**) Average number of attributable heat stress days (WBGT > 28°C) experienced per year by an average member of each age group. Attributable days are calculated for present-day warming levels from reanalysis products (A to C) and models (D to F). Error bars show the median and range across datasets/models in (A), (B), (D), and (E) and median and interquartile range (IQR) across datasets/models in (C) and (F). Note that *y*-axis scales are different between reanalyses and models.

Combining our estimates of attributable days with spatially explicit demographic data (see Materials and Methods), we find that, at the global level, younger generations are disproportionately exposed to a greater number of attributable heat stress days. For example, we find that 583 ([497 to 592]) million children aged 0 to 9 years are globally living through at least 20 attributable heat stress days per year compared to 190 ([166 to 196]) million people aged 60 to 69 years. This means that 3 ([3 to 3.1]) times more younger people compared to older people are experiencing this additional amount of heat stress exposure (Fig. 2A).

The greater exposure of young people in absolute terms arises because of two factors: First, there are more children aged 0 to 9 years (∼1.3 billion) than people aged 60 to 69 years (∼0.6 billion) in the world, and, second, younger people tend to live in regions with higher baseline levels of heat stress and stronger increases in heat stress attributable to human-induced climate change. Even after accounting for differences in global cohort sizes, young people remain more exposed to attributable heat stress. We estimate that 44% ([38 to 45%]) of children aged 0 to 9 years are living through at least 20 attributable heat stress days per year compared to 30% ([26 to 31%]) of the 60- to 69-year cohort, corresponding to a 1.4 ([1.4 to 1.5]) times larger proportion of the younger age group (Fig. 2B).

Estimating the number of attributable heat stress days per year that an average member of each age group lives through at present-day warming levels, a similar pattern emerges. We find that an average (mean) member of the 0- to 9-year age group is expected to experience 28 ([22 to 29]) attributable heat stress days per year. This is 9 ([6 to 9]) more days per year than an average member of the 60- to 69-year cohort (19 [15 to 20] days per year) (Fig. 2C).

We find that young people are disproportionately exposed to both total and attributable heat stress and that the disparity between age groups is slightly higher for attributable heat stress than for total heat stress (fig. S4). For instance, individuals aged 0 to 9 years experience 46% ([41 to 50%]) more attributable heat stress days than those aged 60 to 69 years compared to 33% ([30 to 34%]) more total heat stress. This indicates that younger populations are concentrated not only in regions with high baseline heat stress but also in regions where anthropogenic increases in heat stress are particularly strong.

These comparisons quantify differences in exposure rather than vulnerability. While older adults typically exhibit higher relative mortality risks, children face distinct direct and indirect heat-related risks. Our results describe demographic disparities in exposure rather than health outcomes. This disparity in exposure reflects the alignment of spatial patterns in heat stress, which is the highest in the tropics, with differences in population age structures (figs. S4 and S5). Namely, populations in low-latitude regions are younger, meaning that attributable heat stress overall disproportionately affects younger populations, both in absolute terms and relative to their global population share.

### Robustness of results

We consistently find younger age groups to be disproportionately exposed across a range of heat metrics and thresholds, number of attributable days per year considered, age group comparisons, data products, and attribution methods. There is ongoing research to understand which heat metrics are most relevant for impacts on public health, and results vary locally and across individual studies (*45*), so we test the sensitivity of our results to different metrics and thresholds. Younger age groups are disproportionally exposed independent of whether we use WBGT as a metric to represent humid heat (figs. S6, A to D; S7, A to D; and S8, A to D) or daily maximum temperature as a metric to represent dry heat (figs. S6, I and J; S7, I and J; and S8, F to H). Moreover, the pattern holds when defining heat stress days on the basis of a higher ISO standard WBGT threshold of 30°C, representing high heat stress (figs. S6B, S7B, S8B, and S9, B, E, and G) (*43*, *44*). This pattern also holds when using percentile-based definitions, such as the exceedance of the 90th, 95th, and 99th percentiles of the preindustrial distributions of WBGT (figs. S6, C and D; S7, C and D; S8, C to E; and S9G) or daily maximum temperature in each location (figs. S6, I and J; S7, I and J; S8, F to H; and S9, C, F, and G). This is particularly relevant because percentile-based thresholds can account for the acclimatization of individuals to their local climate more than absolute thresholds (*36*).

Furthermore, while we have shown results comparing people living through at least 20 attributable heat stress days per year (Fig. 2, A and B), the difference between age groups holds when looking at different numbers of attributable days per year (figs. S6, S7, and S9) and generally increases when comparing exposure to higher numbers of attributable days (fig. S9).

We have highlighted results comparing the exposure of the 0- to 9-year age group with the 60- to 69-year age group, broadly corresponding to children today and the generation of their grandparents. Children are more exposed than any other age group, and the difference is progressively more extreme the older the age group children are compared to (Fig. 2). For example, per capita, children aged 0 to 9 years face on average 5 more attributable heat stress days per year compared to the 40- to 49-year age group, 12 more days compared to the 70- to 79-year age group and 14 more days compared to the 80- to 89-year age group (WBGT above 28°C, median across reanalyses; figs. S8A and S9G). As progressively older age groups are considered, the geographic contrast in age structures increasingly drives disparities in exposure, with the oldest populations concentrated in higher-latitude regions and younger populations in more exposed lower latitudes.

### GCM-based attribution

To test robustness to the dataset and statistical method used and to have multiple lines of evidence, we carry out attribution analyses using three different reanalysis products and 10 bias-adjusted global climate model (GCM) simulations. For attribution based on GCM simulations, we apply an empirical nonparametric approach that compares the occurrence of heat stress days (WBGT > 28°C) during a preindustrial baseline period (1850 to 1900) with that during a 30-year period that corresponds to present-day warming levels using historical simulations and projections (see Materials and Methods and note S1).

When comparing results from reanalyses and climate models, the same overall pattern emerges (Fig. 2, note different *y*-axis scales). In both, we find a greater number of absolute and attributable heat stress days in the lower latitudes (figs. S2 and S3) and a corresponding disproportionate exposure of younger age groups (figs. S6 to S8). Nonetheless, at a global level, climate model–based estimates of the number of attributable heat stress days are higher than reanalysis-based estimates, driven by higher estimates in the tropics, particularly in Northwestern South America, Central Africa, the Horn of Africa, and East and Southeast Asia (fig. S13), leading to correspondingly higher estimates of the number of people exposed to attributable heat stress (Fig. 2D and fig. S6). For instance, from climate models, we estimate that 687 ([595 to 837], median and range across models) million children aged 0 to 9 years are exposed to at least 20 attributable heat stress days per year compared to 240 ([201 to 356]) million people aged 60 to 69 years (Fig. 2D). We estimate the younger age group to be on average experiencing 38 ([29 to 43]) days per year compared to 27 ([23 to 33]) experienced by the older age group, corresponding to 10 ([6 to 13]) more days per year for the younger age group (Fig. 2F).

To understand the difference between reanalysis- and GCM-based attribution results, we decompose the contribution of (i) the different statistical methods used, as well as (ii) differences in trends and (iii) variability between GCMs and reanalysis (fig. S13 and note S1). We find the dominant driver to be that GCMs overestimate trends in WBGT in the tropics, explaining on average 85% ([51 to 95%], median and range across GCMs) of the difference with reanalysis-based estimates worldwide. Differences in method explain 15% ([−4 to 48%]) of the difference, with the nonparametric method used for GCMs leading to higher estimates of attributable heat stress days at lower latitudes. Differences in variability lead to smaller and more localized differences (fig. S13). Overall, we find that absolute numbers on the exposure of people to heat stress globally are higher in GCM-based attribution because of higher estimated increases of heat stress in highly populated areas in the tropics. Nonetheless, we find that multiplication factors comparing age groups agree with those obtained from reanalyses (figs. S9 and S10) and that results from GCMs and reanalysis share the same overall pattern of demographic disproportionality (Fig. 2).

### Intergenerational disproportionality persists under near-term warming

**Fig. 3.**
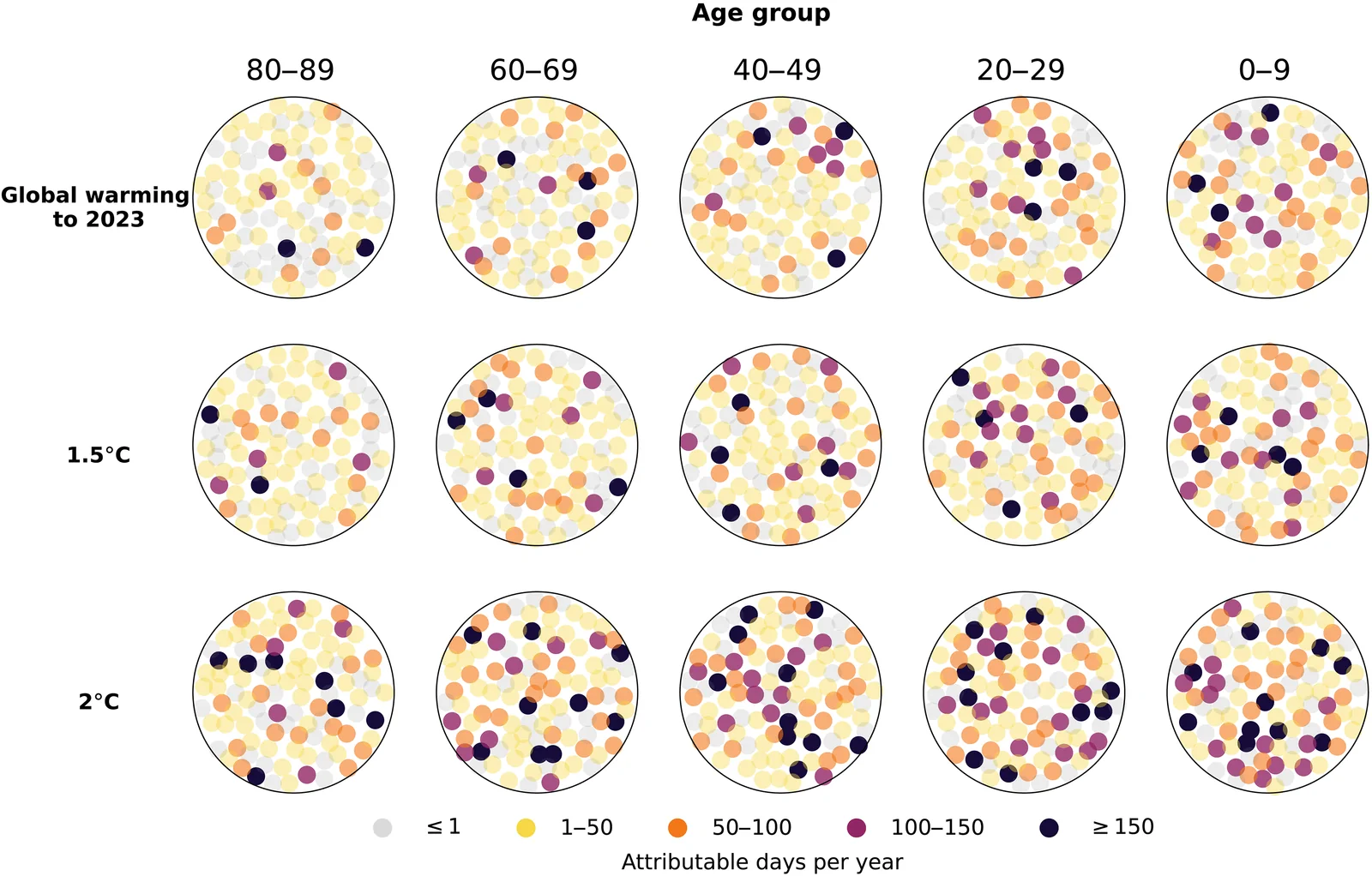
Intergenerational disproportionality persists under near-term warming. Proportion of each age group globally living through different numbers of attributable heat stress days per year, defined as WBGT > 28°C, at present-day warming (up to 2023) and 1.5° and 2°C of warming. Results are shown for the multimodel median across 10 GCMs. Each dot represents 1% of the age group. The 1.5° and 2°C warming levels are identified in each model as the periods in which average 30-year warming crosses 1.5° and 2°C, respectively. Corresponding demographic data are taken from the central year of a 30-year period in which the average warming of a marker SSP2 and RCP4.5 (SSP2-RCP4.5) crosses 1.5° and 2°C, respectively (see Materials and Methods and table S1).

A crucial question is how this intergenerational disproportionality could evolve with additional warming. To this end, we repeat the analysis for near-term warming of 1.5° and 2°C on the basis of GCMs, estimating exposure to attributable heat stress from each GCM during the period when it reaches the respective warming level (Fig. 3 and table S1). To account for demographic changes, we use population and cohort size data corresponding to the time in which a marker scenario projects crossing 1.5° and 2.0°C under Shared Socioeconomic Pathway 2 (SSP2), respectively, in the 2030s and in the 2060s (see Materials and Methods). Under SSP2, a middle-of-the-road socioeconomic scenario, global population is projected to peak at more than 9 billion in the second half of the 21st century, with most growth occurring in low-latitude regions, particularly Sub-Saharan Africa, where age structures mature but still remain younger than in aging high-latitude populations experiencing demographic stagnation or decline (*46*, *47*).

In a 1.5°C world, 4% of the 0- to 9-year age group (51 million children) are projected to experience 150 or more attributable heat stress days per year, up from 3% (40 million children) under present-day warming. In a 2°C world, this fraction further increases to 10% (115 million children; Fig. 3, last column, black dots). Likewise, the exposure of all age groups to heat stress will increase (Fig. 3). In contrast, the number of people experiencing little to no attributable heat stress decreases (Fig. 3, light gray dots).

Despite the increase in exposure across all age groups, children are projected to remain relatively more exposed than other age groups under 1.5° and 2.0°C warming. At present-day warming levels, 11% of 0 to 9 year olds experience 100 or more attributable heat stress days per year compared to only 6% of 60 to 69 year olds (Fig. 3, second and last columns, purple and black dots). Under 1.5° and 2°C warming, this rises to 13 and 23% of 0 to 9 year olds and 9 and 17% of 60 to 69 year olds, representing respectively 1.5 and 1.3 times greater proportions of younger people than older people.

Although young people remain disproportionately exposed under assessed warming scenarios of 1.5° and 2.0°C and across SSP1, SSP2, and SSP3 (table S2), the relative disparity between age groups decreases over time. Even as total exposure increases, the proportional gap between younger and older age groups generally narrows relative to the present day (table S2). For example, at present-day warming levels, the proportion of children aged 0 to 9 years experiencing 100 or more attributable heat stress days is 3.1 times that of adults aged 60 to 69 years compared to 2.3 times at 1.5°C and 1.4 times at 2.0°C (table S2 and fig. S15). Future patterns result from the combined effects of continued global warming and demographic changes, including both changes in global population size and age structures. Namely, further warming leads to an expansion of areas affected by high levels of heat stress (fig. S15, A to C) and increases the fraction of the global population exposed (fig. S15D). This does not produce a saturation effect in which the entire global population is exposed to the same levels of heat stress, as the most extreme increases in heat stress remain geographically confined to lower latitudes. At the same time, projected population growth in lower latitudes increases the fraction of global population exposed to high levels of heat stress across all age groups (fig. S15, E and F). Simultaneously, population aging in regions exposed to heat stress reduces age-based disparity in exposure and increases relative exposure among adults aged 30 years and over, as a growing share of individuals in these regions belongs to older age groups compared to today (fig. S15, E and F). The combined effect of warming and population growth therefore increases overall population exposure beyond what would result from individual factors alone, while population aging reduces age-based disparity in exposure between children and adults (fig. S15, E and F). The decline in age-based disparity does not imply reduced heat-related risks. Rather, larger proportions of all age groups are projected to be exposed to high levels of heat stress. This is especially concerning for adults over 65 years, who face elevated heat-related health risks and are projected to represent an increasing share of the population exposed to high levels of heat stress under continued warming, and for populations with limited adaptive capacity.

## DISCUSSION

Our results show that exposure to total and attributable heat stress is concentrated in lower latitudes and that globally young people are disproportionately exposed compared to older age groups because of the spatial patterns of heat stress and demographics. These findings are in line with previous research showing that tropical and lower-income regions are more exposed to attributable heat extremes [e.g., (*36**,* *39**,* *41**,* *42*)].

Children in most affected areas, predominantly tropical lower and middle-income countries, are likely to be socioeconomically more vulnerable and affected by heat via direct, heat strain–related, and indirect pathways. The indirect pathways through which heat particularly affects children in LMICs include increased diarrheal disease risk (*29*, *30*, *48*), changes in vector-borne diseases (*27*, *28*, *49*), compromised child feeding practices (*25*, *26*), and negative birth outcomes (*50*). These indirect pathways are likely to dominate the child health burden in the regions where attributable exposure to heat stress is the highest (*21*), such as South and Southeast Asia and West Africa. There is also a growing body of evidence on increased heat-related mortality in young (0 to 4 years) children in sub-Saharan Africa (*12*, *32*).

With continued warming, heat stress exposure will increase across all age groups. Studies examining physiological limits of human exposure to humid heat stress under climate change project the regular crossing of these limits in hotspot locations in the world with continued warming (*51*, *52*) and find that such limits are already exceeded locally for short periods today (*53*, *54*). As younger cohorts live and age in a warmer world, they will face higher lifetime exposure to heat, as well as other climate risks (*55*).

Our findings have important implications for intergenerational equity and climate justice. Children have contributed negligibly to historical greenhouse gas emissions yet are disproportionately exposed to both total and attributable heat stress today. Moreover, younger cohorts will also experience greater cumulative exposure to heat and other climate risks over their lifetimes (*55*) while having had no agency in past emission decisions.

The asymmetry in heat stress exposure compounds existing age-related and socioeconomic vulnerabilities, particularly in lower-latitude LMICs where adaptive capacity is limited (*8*). Structural determinants of vulnerability and adaptive capacity, including income, access to health systems, housing quality, and governance, vary geographically (*56*). Regions that emerge in our analysis as having the highest levels of attributable stress and the largest number of people exposed, such as South and Southeast Asia and West Africa (figs. S4 and S5), face such challenges. Many countries in these regions have experienced rapid and unplanned urbanization and the development of informal settlements, leading to inadequate housing conditions that offer limited protection from heat and limited adaptive options (*57*–*59*). South Asia and Western Africa have large shares of their populations used in agriculture, and thus exposed to high levels of outdoor heat stress, and a higher sensitivity to climate-related shocks to food production (*57*, *60*, *61*). Moreover, sub-Saharan Africa has the highest share of people living below the extreme poverty line globally and a high preexisting burden of disease (*8*, *62*). Climate change–induced increases in heat stress therefore exacerbate risks in regions with high levels of socioeconomic vulnerability and limited adaptive capacity.

Our findings underscore the ethical imperative of mitigation and adaptation to advance intra- and intergenerational equity and protect children’s rights (*63*–*66*). Coordinated mitigation efforts consistent with the Paris Agreement temperature goal of limiting warming to 1.5°C would reduce heat stress and other climate-related risks for present and future generations, particularly in the low-latitude regions identified here as exposure hotspots, including West Africa, South Asia, and Southeast Asia.

At the same time, adaptation strategies tailored to these settings are urgently needed to reduce near-term health risks, including for children. Evidence from South Asia suggests that heat health action plans that combine early warning systems, outreach via health professionals, and cooling centers in public spaces can reduce heat-related mortality during extreme heat events (*67*, *68*). In densely populated urban areas, relatively low-cost demand-side interventions, including school-based heat education, adjusted school and work schedules, hydration campaigns, neighborhood outreach, and heat-risk awareness programs, have shown effectiveness because they can be implemented rapidly and reach vulnerable populations with limited access to cooling technologies (*69*). These approaches may be especially relevant in tropical LMIC settings where electricity access and household air conditioning remain limited.

Supply-side interventions also play an important role but face greater infrastructure and equity constraints. In South and Southeast Asia, reflective roofing materials (*67*, *68*), passive ventilation, urban greening, and access to shaded public cooling spaces have been associated with reductions in indoor and neighborhood heat exposure. Although air conditioning uptake is growing in LMICs, particularly in South and Southeast Asia, access remains limited, highly unequal, and insufficient to protect outdoor workers, informal settlements, and low-income households who face the greatest heat exposure (*70*–*72*). Expanding cooling infrastructure without parallel investments in affordable energy access and climate mitigation may also increase the energy demand and inequalities.

The geographic concentration of exposure identified in this study highlights the need for regionally tailored adaptation strategies. In West Africa and other parts of sub-Saharan Africa, where health action plan implementation remains limited compared with South and Southeast Asia, priority actions include strengthening meteorological and public health surveillance systems, establishing locally appropriate heat warning mechanisms, integrating heat risk management into maternal and child health services, and expanding community-based outreach before and during heatwaves (*73*–*75*). Given constrained health system capacity in many low-income tropical countries, current evidence suggests that scalable community-based and behavioral interventions may provide the most immediate and equitable benefits for protecting children, while longer-term investments in resilient housing, cooling infrastructure, and health systems remain essential for sustained adaptation (*76*–*79*).

Our findings further indicate that children remain globally disproportionately exposed under global warming of 1.5° and 2°C, although the relative disparity between age groups narrows over time because of aging populations. This narrowing does not imply reduced risk. Rather, exposure increases across all age groups, including for children and older adults (>65 years), both categories that are particularly vulnerable to heat-related health impacts (*8*).

While reanalysis- and GCM-based results agree in terms of overall spatial pattern and demographic disproportionality, the magnitude of attributable heat stress is higher in the GCM-based estimates. Our decomposition suggests that this difference is primarily driven by stronger simulated trends in tropical WBGT in GCMs relative to reanalysis. The underlying causes of this divergence could be further investigated and may reflect differences in the representation of temperature (*80*) or humidity trends; uncertainties in reanalysis data, particularly in early decades (*81*); and internal variability. Internal variability is generated within GCMs and therefore contributes to intermodel spread but is not sampled in the reanalysis-based estimates, which represent a single realization of the historical climate. Our uncertainty ranges reflect the spread across models or reanalysis products, which are ensembles of opportunity rather than systematically sampled distributions, and therefore capture structural differences between models and datasets but do not constitute a full probabilistic uncertainty estimate. We therefore interpret reanalysis products as our best estimate of the observed climate over the past century and climate models as physically consistent realizations of possible weather and climate states and present results from both as complementary lines of evidence.

Our analysis presents limitations that could be addressed in future studies. First, our analysis focuses on exposure to heat stress rather than health outcomes. Equal WBGT exposure does not imply equal health risk across age groups, as quantifying impacts would require age-specific dose-response relationships, which remain an important area for future research. While the WBGT threshold for moderate heat stress exposure we focus on (WBGT > 28°C) is widely used in the labor and health literature (*82*, *83*), it does not correspond to a uniform physiological effect across individuals, as responses to heat stress vary with factors such as age, health status, clothing, activity levels, and personal acclimatization (*9*, *84*), and as heat can affect health through both direct and indirect pathways. Nevertheless, we demonstrate that the identified patterns of age-based disproportionality are robust across alternative thresholds, including percentile-based definitions that implicitly account for local acclimatization (figs. S6 to S10). Furthermore, our primary metric, indoor WBGT (WBGTi), assumes shaded and well-ventilated conditions (see Materials and Methods). As such, our exposure estimates may be conservative, particularly for populations spending substantial time outdoors, such as outdoor workers or children. This means that the true age-based disparity between younger and older populations is likely to be larger than reported and that our results may be considered conservative. Future work could examine exposure to sunlit conditions and consider the behavioral patterns of specific subpopulations in greater detail.

Moreover, our results rely on gridded demographic projections that assume constant age-group distributions within countries, potentially masking subnational heterogeneity in exposure. Sensitivity analyses using alternative gridded age-structure data suggest that global disparity patterns are robust, although absolute exposure levels vary (fig. S16), reflecting differences in underlying total population distributions in the data products. Last, reanalysis products present uncertainties particularly in early decades and in data-sparse regions, meaning that there are uncertainties in reanalysis-derived trends.

Overall, our results show that increased exposure to humid heat stress attributable to anthropogenic climate change is already occurring today and disproportionately affects younger age groups worldwide. Our findings add to a growing body of evidence on the unequal distribution of consequences from anthropogenic climate change. In particular, our work reveals a global age-based asymmetry in exposure to heat stress and highlights the need for mitigation and adaptation policies to protect children under present and near-term warming.

## MATERIALS AND METHODS

### Indoor WBGT calculation

The WBGT is a heat stress metric originally developed to prevent heat illness in military contexts (*85*) and now standardized by ISO for occupational safety (*43*, *44*). It has been found to be among the best predictors of heat strain compared to other heat stress indices (*86*) and is increasingly applied in epidemiological studies (*82*, *87*, *88*) and assessments of labor productivity under climate change (*83*, *89*, *90*). WBGT is defined as

$$\text{WBGT}=0.7T_{\text{nw}}+0.2T_{g}+0.1T_{a}$$ (1)

where *T*_nw_ is the natural wet bulb temperature, *T*_g_ is the black globe temperature, and *T*_a_ is the dry bulb temperature. The wet bulb temperature is the temperature to which an air parcel can cool via evaporation until it becomes saturated, with latent energy supplied by the air parcel itself. In human heat stress applications, it is interpreted as the theoretical minimum skin temperature achievable through maximal evaporative cooling under idealized conditions (high sweating efficiency, shade, strong air movement, and unrestricted water intake) (*9*, *11*). As these conditions are rarely met, this is a theoretical lower bound of heat stress (*51*–*53*). The natural wet bulb temperature (*T*_nw_) is measured with a sensor freely exposed to the environment, incorporating the effects of wind and radiation, whereas the psychrometric (or thermodynamic) wet bulb temperature (*T*_w_) is defined under conditions of efficient forced ventilation (aspiration) and depends only on temperature and humidity (*91*, *92*). The black globe temperature (*T*_g_) is the temperature measured by a thermometer located at the center of a dark, thermally conductive sphere exposed to the environment (*91*) and represents combined radiant heat load from sunlight and nearby objects. In its full form, the WBGT metric considers the combined effects of temperature, humidity, wind speed, and radiation on heat stress (*91*, *93*).

In this study, we use the so-called WBGTi, a simplified formulation that approximates WBGT under shaded and well-ventilated conditions (*92*) and has been widely applied in previous large-scale studies (*40*, *83*, *90*, *94*–*98*). WBGTi is calculated by approximating *T*_nw_ with *T*_w_ and approximating *T*_g_ with ambient air temperature (*T*_a_), assumptions that are approximately met when excluding the effects of radiation and under sustained moderate to strong wind speeds (>3 m/s) (*92*, *99*).

$$\text{WBGTi}=0.7T_{w}+0.3T_{a}$$ (2)

When derived from outdoor climate variables, WBGTi does not represent indoor thermal environments or building heat dynamics and does not account for mitigation measures such as air conditioning. Rather, it represents an approximation of outdoor heat stress under sheltered or shaded conditions (*40*).

We calculate daily maximum WBGTi from daily maximum temperature, daily mean specific humidity, and daily mean surface pressure, following (*83*, *94*), using climate model output and reanalysis datasets from the Inter-Sectoral Impact Modelling Intercomparison Project (ISIMIP). The wet bulb temperature (*T*_w_) is calculated using the adiabatic NEWT (Noniterative Evaluation of Wet-bulb Temperature) method, which has been shown to be more accurate than other methods (*100*).

We use WBGTi for population-level analysis because (i) it does not require outgoing shortwave and longwave radiation, which is not available in the ISIMIP framework; (ii) by assuming shaded conditions, it avoids making assumptions about local solar exposure, which would depend on local building characteristics or natural infrastructure; and (iii) it can be calculated from daily climate data, avoiding the need for empirical reconstructions of subdaily radiation and wind variability, which cannot be robustly constrained from daily data available through ISIMIP. Moreover, (iv) as discussed in previous detection and attribution studies (*40*), the WBGTi formulation focuses on thermodynamic variables (temperature and humidity), for which anthropogenic influences are well understood and physically grounded, rather than analyzing trends in variables, such as wind speed and sunlight hours, where anthropogenic influences are less clear, well constrained, or globally consistent. Last, (v) WBGTi, formulations similar to ours but accounting for local wind speeds, has been found to rank among the best predictors of heat strain (*86*). We consider the assumption of well-ventilated conditions justified for a large-scale global study in the absence of data on subgrid ventilation while acknowledging that WBGTi may underestimate heat stress under low wind or sunlit conditions.

We use ISO standard absolute WBGT thresholds (*43*, *44*, *101*) to define heat stress days. We focus on the WBGT threshold of 28°C, which indicates moderate heat stress for acclimatized individuals (*43*, *101*). We additionally evaluate our results for a WBGT threshold of 30°C, indicating high heat stress for acclimatized individuals (*43*, *101*), and for percentile-based thresholds, namely the 90th, 95th, and 99th percentiles of WBGT and, separately, for daily maximum temperature, calculated from the full annual distribution in a preindustrial (1850 to 1900) climate. This approach avoids the use of rolling or seasonally defined percentiles while implicitly focusing on the warm season through exceedances of the upper tail of the annual distribution. ISO thresholds are defined for occupational settings but are calibrated to set limits on safe activity levels classified by metabolic rate (*101*), which can be generalized to a broad range of activities (*51*). Similar thresholds are used across studies in physiology, sports science, and epidemiology and for different age groups (*88*).

By design, the assumption of shaded conditions causes WBGTi to underestimate heat exposure relative to outdoor WBGT under direct solar radiation (*89*). Our analysis therefore characterizes heat stress under sheltered conditions and does not capture peak heat stress amplified by solar exposure (*40*). This can particularly affect children, who generally spend more time outdoors than adults, or outdoor workers. While the same ISO thresholds are recommended for indoor and outdoor WBGT (*101*), our results should be interpreted as conservative for populations exposed to direct sunlight, low wind conditions, or nonacclimatized individuals. This motivates our focus on moderate and high WBGT thresholds (28° and 30°C), excluding the very high or “critical” (*43*) WBGT threshold of 33°C. Although WBGTi can underestimate extreme values relative to formulations that explicitly account for radiation and wind (*102*), it has been shown to outperform other commonly used approximations of WBGT such as simplified WBGT (*93*, *102*) and remains a widely used indicator for population-level and attribution analyses in large-scale studies (*40*, *83*, *90*, *94*, *96*–*98*). Sensitivity analyses using outdoor WBGT could be considered for selected locations, but WBGTi remains appropriate for large-scale assessments given ISIMIP data constraints, with results to be interpreted as conservative for outdoor exposure, and extreme heat stress conditions.

We calculate WBGTi from three observation-based reanalysis products made available within Phase 3a of the Inter-Sectoral Impact Modelling Intercomparison Project (ISIMIP3a), described by Frieler *et al.* (*103*) and references therein. These products are GSWP3-W5E5, 20CRv3-W5E5, and 20CRv3-ERA5, available for the period of 1901 to 2019 at daily 0.5° resolution (*104*). These products combine centennial reanalysis datasets for the early 20th century with ERA5-based data from 1979 onward, harmonized within ISIMIP3a for use in validation of impact models and attribution of observed impacts (*103*).

We also calculate daily WBGTi from the output of 10 GCMs that are part of Coupled Model Intercomparison Project Phase 6 (CMIP6), previously bias adjusted against W5E5 for the reference period of 1979 to 2014 and statistically downscaled to a common 0.5° resolution using ISIMIP3BASD version 2.5.0 within ISIMIP3b (see table S3) (*105*–*107*). We use output from simulations with transient historical natural and anthropogenic forcings for the period of 1850 to 2014, combined with the SSP3 and Representative Concentration Pathway 7.0 (SSP3-RCP7.0) scenario from 2015, which is the middle-of-the-road scenario available in ISIMIP3b. Using bias-adjusted and downscaled GCM data makes it possible to interpret model results on impact-relevant absolute magnitude thresholds as well as percentile-based thresholds and at a higher spatial resolution than native model grids.

### Attribution based on reanalysis data

We carry out attribution on reanalysis data using nonstationary statistical distribution modeling, taking inspiration from methods used in extreme event attribution (*108*). Namely, independently for each grid cell and calendar month, we fit daily WBGTi data to a nonstationary normal distribution, where the location parameter (mean, μ) is allowed to vary as a linear function of a covariate representing anthropogenic climate change, while the scale parameter (variance, σ^2^) is assumed to remain constant.

As is common in extreme event attribution (*108*), we use GMST as a covariate, obtained from Forster *et al.* (*109*), smoothed using loess with a 4-year window (GMST′) to reduce the influence of modes of climate variability such as El Niño–Southern Oscillation. GMST is commonly used as a covariate in nonstationary modeling for extreme event attribution to represent how local climate variables respond to global warming (*108*). This approach is based on the assumption that the dominant changes in the distribution are driven by anthropogenic warming, rather than by internal variability or other forcings (*108*). Conceptually, the use of GMST derives from pattern-scaling methods and from studies showing that regional climate responses across a range of indicators scale approximately with changes in global mean temperature (*110*, *111*).

The resulting statistical model is

$$\begin{array}{c} X_{i,j}∼N\left(\mu_{i,j},{\sigma^{2}}_{j}\right)\\ \mu_{i,j}=\beta_{0,j}+\beta_{1,j}\cdot {\text{GMST}}_{i}^{\prime } \end{array}$$ (3)

*X_i,j_* denotes daily WBGTi values in year *i* and month *j*, GMST′*_i_* corresponds to the smoothed GMST anomaly in year *i*, β_0,*j*_ and β_1,*j*_ are month-specific regression coefficients, and σ*_j_* is the month-specific standard deviation, assumed constant in time.

The model is estimated independently for each month, yielding three parameters per month (σ*_j_*, β_0,*j*_, and β_1,*j*_) and, thus, a total of 36 parameters for each grid cell. GMST′ is identical across months, but regression coefficients are estimated separately for each monthly model.

Model parameters were estimated using maximum likelihood, using the dist_cov Python package (*112*, *113*). Convergence was assessed using the optimizer’s convergence flag and by verifying that all estimated standard deviations were strictly positive and finite. Convergence failures occurred only for one reanalysis dataset (GSWP3-W5E5), affecting 36 individual grid cell-month fits of more than 9.3 million total fits (3 datasets × 12 months × 259,200 grid cells), corresponding to less than 0.00039% of all fits. Grid cell-month combinations where convergence failed were excluded from subsequent analyses. Given their negligible frequency, excluding these cases does not affect aggregate results.

We then define two theoretical distributions of WBGT for each month, one in the present-day climate, based on the 2023 value of GMST′, and one in a preindustrial climate, based on the 2023 value of GMST′ minus the global warming index (GWI), as often done in extreme event attribution to represent a preindustrial climate (*4*, *5*, *108*). The GWI is a measure of human-induced global warming since the reference period of 1850 to 1900 (*109*, *114*, *115*), meaning that subtracting this from the GMST covariate allows us to model the distribution of the variable in the reference preindustrial period. We use the best estimate of anthropogenic warming from the GWI in 2023 from Forster *et al.* (*109*), i.e., 1.29°C, rounded to 1.3°C in this analysis, and test the sensitivity of our results to uncertainty in the GWI (see note S1). From these distributions, we estimate the daily probability of exceeding the heat stress threshold (T) in the present-day climate (*p*_1_) and in a preindustrial climate (*p*_0_) for each month of the year

$$\begin{array}{c} p_{1,j}=P\left(X_{j}>T|\;{\text{GMST}}^{\prime }={\text{GMST}}_{2023}^{\prime }\right)\\ p_{0,j}=P\left(X_{j}>T|\;{\text{GMST}}^{\prime }={\text{GMST}}_{2023}^{\prime }-\text{GWI}\right) \end{array}$$ (4)

We then quantify the number of attributable heat stress days (nAHD) expected to cross the threshold because of anthropogenic climate change for each month (nAHD*_i_*) on the basis of the difference in these exceedance probabilities and the length of the month, ignoring leap years (*n*_days,*j*_), and sum the result across all months to quantify the attributable number of heat stress days per year (nAHD_year_)

$$\begin{array}{c} {\text{nAHD}}_{j}=\left(p_{1,j}-p_{0,j}\right)\cdot n_{\text{days},j}\\ {\text{nAHD}}_{\text{year}}=\sum_{j=1}^{12}\left({\text{nAHD}}_{j}\right) \end{array}$$ (5)

Attribution is performed independently for each grid cell, meaning that, in locations with sparse exceedances of the heat-stress threshold, probability estimates are more uncertain and can be conservative, leading to increased pixel-scale noise relative to spatially pooled approaches. However, such locations contribute relatively little to aggregated nAHD estimates, limiting their influence on the overall results. Moreover, as the attribution framework models nonstationarity primarily through changes in the mean, this component dominates the aggregated attribution signal.

To account for uncertainties in the underlying reanalysis data, we fit the same statistical model across three different observation-based reanalysis products provided in ISIMIP3a. The spread across the different data products gives us an estimate of uncertainty in our observational line of evidence resulting from dataset uncertainties but does not explicitly include natural variability or parametric uncertainty, which would make our uncertainty intervals in the reanalysis-based estimates larger. To include the effects of natural variability, we complement this line of evidence with a GCM-based line of evidence.

We define two statistical distributions corresponding respectively to the present climate and a preindustrial climate by subtracting the GWI from the GMST covariate to define the preindustrial climate. Using present-day GMST minus GWI allows to extrapolate past the period of available reanalysis data, allowing to compare results against a common preindustrial (1850 to 1900) baseline for both reanalysis and GCM attribution results (*108*). This means that the time period of reanalysis data used for the model fit theoretically does not affect our definition of the preindustrial climate. Nonetheless, decadal variability and spurious trends in reanalysis data can affect the estimated parameters, in particular the slope of the location parameter with respect to GMST (β_1,*j*_, Eq. 3). In extreme event attribution, it is generally advised to use a long, consistent time series of station data to estimate the sensitivity to GMST as robustly as possible (*108*). Nonetheless, reanalysis data assimilates less observations in early decades, meaning that data and trends before the mid-20th century are generally less reliable. A balance therefore needs to be struck between the length of the time series used for the trend estimation and the quality and reliability of the data.

We test the sensitivity of our results to the time period of reanalysis data used by fitting the same nonstationary statistical model on two different time periods of data, namely 1901 to 2019 and 1950 to 2019. In both cases, our preindustrial baseline is defined as GMST′_2023_ − GWI, thus corresponding to the period of 1850 to 1900. We find using different time periods has locally important differences in some reanalysis datasets, in particular GSWP3-W5E5, but that it does not affect our overall results (figs. S11 and S12 and note S1). Given the greater reliability of reanalysis data in later decades, we present main results using parameters from the model fitted to 1950 to 2019 data. Last, we test the sensitivity of our results to the assumption that the variance of WBGT does not change as a consequence of GMST′ by comparing the statistical model described above to one where the standard deviation is also modeled as a linear function of GMST′ and find that this has little impact on the results (figs. S11 and S12 and note S1).

### Attribution based on climate models

For the climate model–based line of evidence, we quantify the change in the frequency of exceedance of heat stress thresholds between a 51-year preindustrial period (1850 to 1900) and a 30-year period centered on present-day warming levels using empirical percentiles, independently for each grid cell, on the basis of historical and SSP3-RCP7.0 simulations. To identify a 30-year period in models matching present-day warming levels, we find the end point of a 10-year moving average of GMST that, for each model, most closely matches the observed warming of GMST [i.e., 1.19°C for 2014 to 2023 from Forster *et al.* (*109*)]. We then take this as the central year of a 30-year window that represents present-day warming levels in each model simulation (table S1). We note that finding a 30-year period that matches the 2023 GWI value (1.3°C) leads to very similar model years being selected. We estimate the probability of exceeding absolute magnitude or percentile-based heat stress thresholds on any given day in the preindustrial period (*p*_0_) and the present-day warming period (*p*_1_) on the basis of their empirical frequency of exceedance in the two samples. We quantify the additional number of days crossing the threshold per year in each location resulting from anthropogenic climate change (nAHD_year_) on the basis of the difference between these two empirical probabilities

$${\text{nAHD}}_{\text{year}}=\left(p_{1}-p_{0}\right)\cdot 365$$ (6)

We account for uncertainty in the model-based line of evidence by carrying out the analysis separately for simulations from one ensemble member each from 10 different CMIP6 models. Uncertainty ranges represent the spread across models, which samples both structural model uncertainty and internal variability. As only one ensemble member per model is used, internal variability is not explicitly isolated from model uncertainty but is included in the reported ranges.

All calculations are performed independently at each grid cell, which can increase pixel-scale noise compared to spatially pooled approaches. In locations with sparse exceedances of the heat-stress thresholds, probability estimates are therefore more uncertain and often conservative. However, these locations contribute relatively little to the aggregated nAHD and thus have limited influence on the overall results. We use GCM data that have been previously bias adjusted and statistically downscaled using ISIMIP3BASD version 2.5.0 within ISIMIP3b (*105*–*107*). Using bias-adjusted climate model output allows us to compare model and observation results using impact-relevant absolute magnitude thresholds and not only thresholds defined on the basis of return periods, as is most common in extreme event attribution (*108*). Comparing the return period of the WBGT 28°C threshold in bias-adjusted models and reanalysis for matching warming levels, we see that models show a reasonable representation or a moderate overestimation of its frequency of exceedance (fig. S17). Moreover, the ISIMIP3BASD method is trend-preserving, conserving the modeled climate change signal in the mean and across quantiles (*107*, *116*). As a result, bias adjustment is not expected to artificially constrain the attribution signal. However, the procedure does not explicitly preserve temporal dependence, spatial coherence, or the exact shape of the distribution tails.

By presenting results from reanalysis and climate models separately, we aim to reflect uncertainty in the attributable signal across two complementary lines of evidence. Historical climate model simulations provide physically internally consistent realizations of the climate but may omit or simplify some real-world processes (e.g., regional aerosol concentrations and changes, local changes in irrigation, land use, atmospheric circulation, etc.) (*80*). Reanalysis products assimilate observations and represent our best estimate of past climate at decadal to centennial scale, but their quality depends on the underlying forecast model and the availability of observations for assimilation. In early decades and in data-scarce regions, observations to assimilate are particularly lacking, meaning that centennial trends in reanalysis products must be interpreted with caution (*1*, *117*). While these two lines of evidence are not fully independent, given that reanalysis systems assimilate, and climate models are evaluated or calibrated against similar observational datasets, they nevertheless provide complementary lines of attribution evidence.

To understand differences between climate model and reanalysis-based attribution results, we decompose the contribution of (i) the different statistical methods used, as well as (ii) differences in trends and (iii) variability between GCMs and reanalysis (see note S1 and fig. S13). First, we test the sensitivity of our estimates to the different statistical methods applied by applying both the shift-fit and empirical percentile methods to the same climate model output and comparing results. Second, we estimate the difference resulting from differences in trends between GCMs and reanalysis by estimating the number of attributable heat stress days from GCMs using the shift-fit method but, in each grid cell, hold the variance estimate equal to the value estimated from one reanalysis product (20CRv3-W5E5) so that the only difference is due to differences in estimated monthly trends in WBGT relative to GMST. We choose 20CRv3-W5E5 as the reference reanalysis product for this analysis as the GCMs were bias adjusted against W5E5 (*107*), and thus can be expected to be more similar to it, and as it shows median results in our analysis between the three reanalysis datasets assessed (Fig. 2 and fig. S3). Third, we estimate the difference resulting from differences in variability between GCMs and reanalysis by estimating the number of attributable heat stress days from GCMs using the shift-fit method but, in each grid cell, for each monthly distribution, hold the trend of the location parameter against GMST equal to the estimate from reanalysis and use an observational GMST time series so that the shift in the location parameter is determined by the shift-fit model applied on reanalysis data. We compare the multimodel mean of GCM results with results from reanalysis and compare results spatially and as zonal averages.

### Demographic data

We produce a gridded demographic dataset that is spatially and temporally explicit by combining gridded population data made available through ISIMIP3a and ISIMIP3b with cohort size data from Wittgenstein Center Human Capital Data Explorer (WCDE) version 2 (*46*, *47*, *118*).

We use methods based on Thiery *et al.* (*55*) but update gridded population data to the more recent datasets available through ISIMIP3a and ISIMIP3b (*103*, *119*, *120*). Reconstructions are available up to 2021 based on HYDE version 3.3 (*121*) and national population estimates from UN World Population Prospects 2019 (*122*), while projections are based on the national SSP scenarios from Lutz *et al.* (*47*) and gridded population projections from the National Center for Atmospheric Research (*123*, *124*). Cohort size data from WCDE are available at a country level for the period of 1950 to 2100 (reconstructions up to 2015 and projections thereafter) expressed for 5-year age cohorts at 5-year time snapshots. We linearly interpolate cohort sizes to exact ages and apply a simple mean-preserving correction to ensure coherence between the values expressed for 5-year age groups and for individual ages and linearly interpolate the 5-year snapshots to yearly values to obtain estimates for each age and each year. We use projections of gridded population and national cohort sizes from the SSP2 scenario, which is a middle-of-the-road socioeconomic scenario. We test sensitivity to this choice for 1.5° and 2°C warming by comparing results with those obtained using demographic data from SSP1 and SSP3.

We combine gridded population data and national cohort size data to obtain spatially explicit demographic data. Following the approach of Thiery *et al.* (*55*), we assume that country-level cohort proportions are equally distributed in space in each country and apply these country-level proportions to the gridded population data using fractional country masks from ISIpedia (see Data, code, and materials availability). One hundred ninety-seven countries are covered by both the WCDE cohort size data and the fractional country masks, leading to >99.94% of the global population in 2023 under SSP2 in the original gridded dataset being conserved after this processing.

We test the sensitivity of our results to the assumption of a constant within-country age distribution by comparing them with results derived from age-sex gridded population data provided by WorldPop (*125*). For both approaches, we use demographic data for the year 2020. WorldPop data, originally available at 1-km spatial resolution, are remapped to a 0.5° grid while conserving total population counts. We do not use the WorldPop-based estimates in our main analysis because these data are only available through 2030 and therefore cannot be used to generate projections under 1.5° and 2°C global warming scenarios.

### Exposure estimation

Thanks to the ISIMIP framework, bias-adjusted climate data and population data are available on the same spatial resolution. We estimate exposure to attributable heat stress days by combining obtained grid scale attribution estimates with spatially explicit demographic data. We identify the number of people of each age group in each grid cell exposed to a given number of attributable days and aggregate globally across each age group to estimate the number of people of the age group exposed, the proportion of the age group exposed, and the number of attributable days experienced by an average member of the age group.

For the estimates based on climate models, we estimate exposure from each model independently and communicate the median and the range across models as our best estimate and our uncertainty range, thus quantifying uncertainties from model differences and natural variability. For our reanalysis-based estimates, we estimate exposure from all three datasets using the fitted distributions. We communicate the median result as our best estimate and the range across the three reanalysis datasets as our uncertainty estimates, quantifying uncertainty from dataset differences.

To evaluate exposure at present-day warming levels, we use demographic data corresponding to the year 2023 in SSP2. To evaluate exposure at 1.5° and 2°C warming levels, we must select representative years from the demographic projections, given that exposure depends not only on changes in heat stress at higher warming levels but also on population size, age structure, and spatial distribution, which are determined by the socioeconomic assumptions underlying the SSPs.

We identify representative years for the demographic data by obtaining global mean warming along an SSP2-RCP4.5 pathway using projections from the IPCC Sixth Assessment Report (AR6) scenario database. This scenario is chosen as it represents a middle-of-the-road pathway in terms of both demographic development (SSP2) and climate mitigation and resulting end-of-century global warming (RCP4.5) (*126*). In particular, we extract the median global mean temperature trajectory of the AR6 marker SSP2-RCP4.5 scenario produced by the MESSAGE-GLOBIOM 1.0–integrated assessment model coupled to the FaIR simple climate model emulator (*127*, *128*). We then identify the central year of the 30-year period during which this trajectory first reaches the 1.5° and 2°C warming levels. For the 1.5°C warming level, this corresponds to 2034, while for the 2°C warming level, this corresponds to 2062.

We calculate exposure at 1.5° and 2°C of global warming using demographic projections from SSP2 corresponding to the years 2034 and 2062, respectively. To attribute changes in exposure between present-day warming and the 1.5° and 2°C warming levels, we perform a stepwise decomposition that isolates the contributions of (i) climate change, (ii) population growth, and (iii) changes in age structure. First, we isolate the role of climate change by allowing heat stress levels to vary according to the respective warming level while holding gridded population counts and age structure fixed at their 2023 values. We then allow the total population in each grid cell to evolve according to SSP2 projections while keeping the age structure (i.e., the proportional distribution of age groups within each grid cell) fixed at 2023 levels. This step isolates the additional exposure attributable to spatial population growth alone. Last, we allow the age structure within each grid cell to evolve according to SSP2 projections. The residual change in exposure relative to the previous step is attributed to demographic aging.

We test the sensitivity to our choice of SSP2-RCP4.5 to select representative years for the demographic data. First, we test how selecting a different RCP pathway influences the year in which 1.5° and 2°C are crossed in the marker scenario and, therefore, the year of demographic data used to calculate future exposure. We note that the choice of RCP has a minimal effect on the year in which 1.5°C is crossed, except for the most ambitious RCP1.9, where 1.5°C is never crossed, while it has a larger effect on the year in which 2°C is crossed (table S2). Second, we test the influence of using SSP3 demographic data, which are based on socioeconomic assumptions of high fertility and slower socioeconomic development, instead of SSP2 demographic data (*46*, *126*). We present results obtained using demographic data and years representative of the warming levels obtained from SSP2-RCP4.5, SSP3-RCP4.5, SSP2-RCP6.0, and SSP3-RCP6.0 and with SSP2 demographics held constant at 2023 levels (table S2).

## Supplementary Materials

### This PDF file includes:

Supplementary Text Figs. S1 to S17 Tables S1 to S3 References
