# Supplementary material for: Age-specific exposure to human-induced increases in humid heat
Source: Sci Adv. 2026 Aug 26;12(35):eaeb3232. doi: 10.1126/sciadv.aeb3232 (PMC13510599; doi:10.1126/sciadv.aeb3232)
Supplement: Supplementary file 1 [file sciadv.aeb3232_sm.pdf]

Science Advances
AAAS

Supplementary Materials for

Age-specific exposure to human-induced increases in humid heat

Rosa Pietroiusti *et al.*

Corresponding author: Rosa Pietroiusti, rosa.pietroiusti@vub.be

Sci. Adv. **12**, eaeb3232 (2026)
DOI: 10.1126/sciadv.aeb3232

This PDF file includes:

Supplementary Text
Figs. S1 to S17
Tables S1 to S3
References

Supplementary Text

Supplementary Note 1.

We use different statistical methods for the attribution of heat stress, based on three reanalyses datasets, and ten global climate models. First, we apply a shift-fit method on reanalysis data, inspired by non-stationary distribution modelling regularly used in extreme event attribution *(108)*. This method is comparable with methods used in observation-based attribution in other emerging global systematic attribution frameworks. For instance, the observational line of evidence in the Climate Shift Index (*37*, *38*) detrends temperature variables from reanalysis at the grid cell level by assuming a skew-normal distribution whose location shifts with global mean surface temperature (GMST). The CSI method models 24 overlapping 30-day calendar periods independently per grid cell, instead of 12 (one per month), as in this study, and similarly to our method model the location parameter of each distribution as a linear function of GMST. Another similar global gridscale method is ATTRICI (*117*), which models non-stationary parametric distributions for many climate variables from reanalysis, in which the location parameter varies continuously as a function of GMST and the calendar day.

The shift fit-based attribution approach makes it possible to create an observation-based counterfactual for the pre-industrial climate even if reanalysis data does not span back to the mid-1800s, and thus provide a semi-observational line of attribution evidence. We use the Global Warming Index (GWI) to modify the covariate value and thus estimate a distribution of daily WBGT for each month that represents a pre-industrial climate, extrapolating past the available time window of data and creating factual and counterfactual distributions that refer to the same baseline, in terms of GMST, as the GCM-based line of attribution evidence (see Methods). We apply the method on reanalysis data for 1950-2019, estimating the trend in location parameter with respect to GMST and an average monthly variance parameter based on this time period of data. We then extrapolate the covariate value, by setting it equal to observed warming in 2023 (previously smoothed to remove natural variability, $GMST'_{2023}$), or to warming in 2023 minus the GWI ($GMST'_{2023}$ – GWI) to represent climate conditions in the pre-industrial (1850-1900) period. The flexibility of the method means that we are can test the sensitivity of our results to (i) using a different time-period of reanalysis data to estimate parameters, and (ii) allowing variance to also vary as a function of GMST (see below).

Secondly, we apply an empirical percentiles-based method on bias-adjusted climate model data from historical and SSP3-RCP7.0 simulations. We compare threshold exceedances during a 30-year window corresponding to 2023 warming levels to a 51-year window corresponding to a pre-industrial climate (1850-1900). This method has the benefit of making no parametric or functional assumptions about the shape and non-stationarity of the WBGT distribution, allows us to include different models and ensemble members with different representations of natural variability and model uncertainties, and adds an additional climate-model based line of evidence which increases the confidence in causal attribution statements compared to basing it exclusively on observation or reanalysis-derived data, which represents only one realization of natural variability and is affected by inconsistencies in early decades of reanalysis data. The overall agreement of these two lines of evidence, both pointing to higher attributable heat stress in lower latitudes and a disproportionate exposure of younger age groups, gives confidence to our attribution results.

To understand differences between climate model and reanalysis-based attribution results, we decompose the contribution of (i) the different statistical methods used, as well as (ii) differences in trends and (iii) differences in variability between GCMs and reanalysis (Fig. S11). First, we test the sensitivity of our estimates to the different statistical methods applied, by applying both the shift-fit and the empirical percentile method to the same climate model output and comparing results. Second, we estimate the difference due to differences in trends between GCMs and reanalysis by estimating the number of attributable heat stress days from GCMs using the shift-fit method but, in each grid cell, hold the variance estimate equal to the value estimated from one reanalysis product (20CRv3-W5E5), so that the only difference is due to differences in estimated monthly trends in WBGT relative to GMST. We choose 20CRv3-W5E5 as the reference reanalysis product for this analysis as the GCMs were bias-adjusted against W5E5 (*107*), thus can be expected to be more similar to it, and as it shows median results in our analysis between the three reanalysis datasets assessed (Fig. 2, Fig. S3). Third, we estimate the difference due to differences in variability between GCMs and reanalysis by estimating the number of attributable heat stress days from GCMs using the shift-fit method but, in each grid cell, for each monthly distribution, hold the trend of the location parameter against GMST equal to the estimate from reanalysis, and use an observational GMST timeseries, so that the shift in the location parameter is determined by the shift-fit model applied on reanalysis data.

We find most of the difference in estimates between reanalysis and climate models can be attributed to differences in trends of heat stress metrics relative to global mean surface temperature in lower latitude land areas. Differences in monthly trends explain 85% [51 – 95%, median and range across GCMs] of differences between the estimated number of attributable heat stress days (nAHD) from GCMs and 20CRv3-W5E5 globally (Fig. S13). GCMs overestimate trends in lower latitude land areas where heat stress levels are high, notably Northwestern South America, Central Africa and Eastern Africa Africa, and Southeast Asia, leading to high absolute estimates of attributable heat stress days in these locations. Less strong overestimations occur in Central and Eastern North America, North Africa, East Asia and Australia. We note that the pattern of difference in trends between GCMs and reanalysis is spatially and temporally varied, with trends both over and underestimated globally, leading, for instance to underestimations in parts of South Asia and West Africa (Fig S13b). Nonetheless, global results are driven by overestimations in lower latitude land areas where exceedances of the heat stress threshold are frequent.

Differences in statistical methodology explain 15% [-4 – 48%] of differences between estimates from GCMs and 20CRv3-W5E5. On average, the non-parametric method used for GCMs leads to higher estimates of attributable heat stress days at lower latitudes, suggesting an increasing skewness of the distribution in these locations not represented by the shift-fit method (Fig. S13d). Differences in variability lead to smaller and more localized differences, contributing only 1% [0 – 7%] of the difference globally.

GCMs both underestimate and overestimate nAHD globally, compared to reanalysis. For instance, total and attributable heat stress days are underestimated by GCMs in parts of South Asia, the Sahel, Western South America, Southern Africa and Western Indonesia (Fig. S13a). Nonetheless, lower latitude land areas where trends are overestimated in GCMs, dominate the global signal when analysing exceedances of an absolute magnitude heat stress threshold, as in our analysis. We decompose what regions drive most of the difference in terms of population exposure and find that the greatest absolute difference between GCMs and models comes from

overestimation of attributable heat stress in highly populated lower latitude areas, particularly in East Asia and Southeast Asia (additional figures are included in the online GitHub repository).

We also test the sensitivity of our reanalysis-based attribution results to the statistical choices made, namely (i) the choice of time period of reanalysis data used for the shift fit, and (ii) the assumption of a time-constant variance, as well as (iii) to the uncertainty in the GWI estimate.

First, we compare our results on the change in heat stress days between present day ($GMST'_{2023}$) and a pre-industrial climate ($GMST'_{2023}$ – GWI) estimated using data from 1950-2019, in our main results, with results obtained when using the full available timeseries of reanalysis data, for the period 1901-2019. Both results compare a present-day climate with a pre-industrial climate, but natural variability in the timeseries can affect the estimated parameters and thus the attribution results.

This leads to relatively minor differences in nAHD estimates, with some over and underestimation (Fig. S11). In terms of zonal averages, differences range between 0 – 2.5 days per year across best estimates of the three datasets, when aggregated in 10º latitude bands, with the highest disagreement found in GSWP3-W5E5. GSWP3-W5E5 shows higher nAHD estimates in Central Africa and Indonesia, lower estimates in India and Southeast Asia, and a mixed signal in South America when using data from 1950-2019 compared to 1901-2019. The other reanalysis products, 20CRv3-ERA5 and 20CRv3-W5E5 show smaller magnitudes of difference with higher nAHD estimates in South America, Indonesia and parts of East, Southeast and South Asia, and lower nAHD estimates in Central Africa and parts of East Asia (Fig. S11).

Second, we compare our estimates when assuming time-constant variance with estimates obtained when we allow variance to vary as a linear function of GMST. The statistical model in this case is thus:

$$X_{i,j} \sim N(\mu_{i,j}, \sigma^2_{i,j})$$
$$\mu_{i,j} = \beta_{0,j} + \beta_{1,j} \cdot GMST'_i$$
$$\sigma_{i,j} = \gamma_{0,j} + \gamma_{1,j} \cdot GMST'_i \qquad \textit{Eq. 1}$$

Where, $X_{i,j}$ denotes daily WBGTi values in year i and month j, $GMST'_i$ corresponds to the smoothed GMST anomaly in year i, $\beta_{0,j}$ and $\beta_{1,j}$ are month-specific regression coefficients that determine changes in the location parameter (mean), and $\gamma_{0,j}$ and $\gamma_{1,j}$ are month-specific regression coefficients that determine changes in the standard deviation. In this specification, both the location and spread of the distribution are allowed to vary linearly with GMST.

This causes generally minor differences in our results (Fig. S11), leading to a slight overestimation of nAHD in lower latitudes, in the order of 0 – 1.5 days per year when aggregated in 10º latitude bands.

In terms of impacts on exposure results, estimates of number and proportion of people exposed are slightly lower when using 1901-2019 data compared to our baseline results, across all age groups, as is the average number of heat stress days experienced per age group. When allowing the variance parameter to change, global aggregated results are similar or slightly higher than our baseline results. Within uncertainty intervals estimates from all three methods overlap. Using our baseline method, we find 3.3 [3.3 – 3.4] more people aged 0-9 exposed to 20

or more attributable heat stress days compared to people aged 60-69, whereas using 1901-2019 data we find a factor of 3.2 [3.2 – 3.3] and using time-varying sigma we find 3.3 [3.2 – 3.5]. We find a 48% [47 – 54%] larger proportion of younger than older people exposed in our baseline method, compared to 43% [42 – 49%] using 1901-2019 data and 48% [44 – 55%] when allowing variance to change. We find young people to be experiencing 9 [7 – 9] more days per capita than older people when using our baseline method, compared to 5 [5 – 9] when using 1901-2019 data and 9 [7 – 10] when allowing variance to change.

Finally, we test sensitivity of our reanalysis-based attribution results to uncertainty in the GWI estimate. We obtain the best estimate and 90% confidence interval of GWI from (*109*), corresponding to 1.29°C (1.16°C - 1.40°C), which we round to 1.3°C (1.2°C - 1.4°C). Using the lower and upper bounds of the confidence interval, we find the best estimate of people aged 0 – 9 exposed to at least 20 attributable heat stress days ranges from 568 – 595 million (full range including dataset uncertainty and GWI uncertainty, 488 – 609 million people), corresponding to 3 times more people compared to people aged 60 – 69 (full range 3 – 3.2 times). We find people aged 0 – 9 exposed to 26 – 30 attributable heat stress days per year (full range 20 – 31 days), or 8 – 10 more days compared to people aged 60 – 69 (full range 6 – 10 days). While accounting for uncertainty in the GWI increases the size of uncertainty intervals in our reanalysis-based results, we find similar overall results to those we obtain when using the best estimate of GWI.

In extreme event attribution, the synthesis of models and observations to obtain a single best estimate of an attributable change, is a theoretically important, albeit challenging, step (*129*). Recent global attribution frameworks have proposed methods to synthesize results from models and observations (*2*, *3*), but have mostly expressed these as probability ratios. We decide to focus on the additional days per year crossing an impact-relevant threshold, due to the tangibility and impact-relevance of this metric. This allows us to highlight locations that are exposed to a high absolute number of heat stress days, and to not overemphasize locations where low pre-industrial probabilities lead to high probability ratios. We present model and reanalysis-based results separately, as we consider them separate and complementary lines of evidence. We show that overall results on demographic disparity agree across the two lines of evidence and decompose the drivers of the differences found between the two lines of evidence.

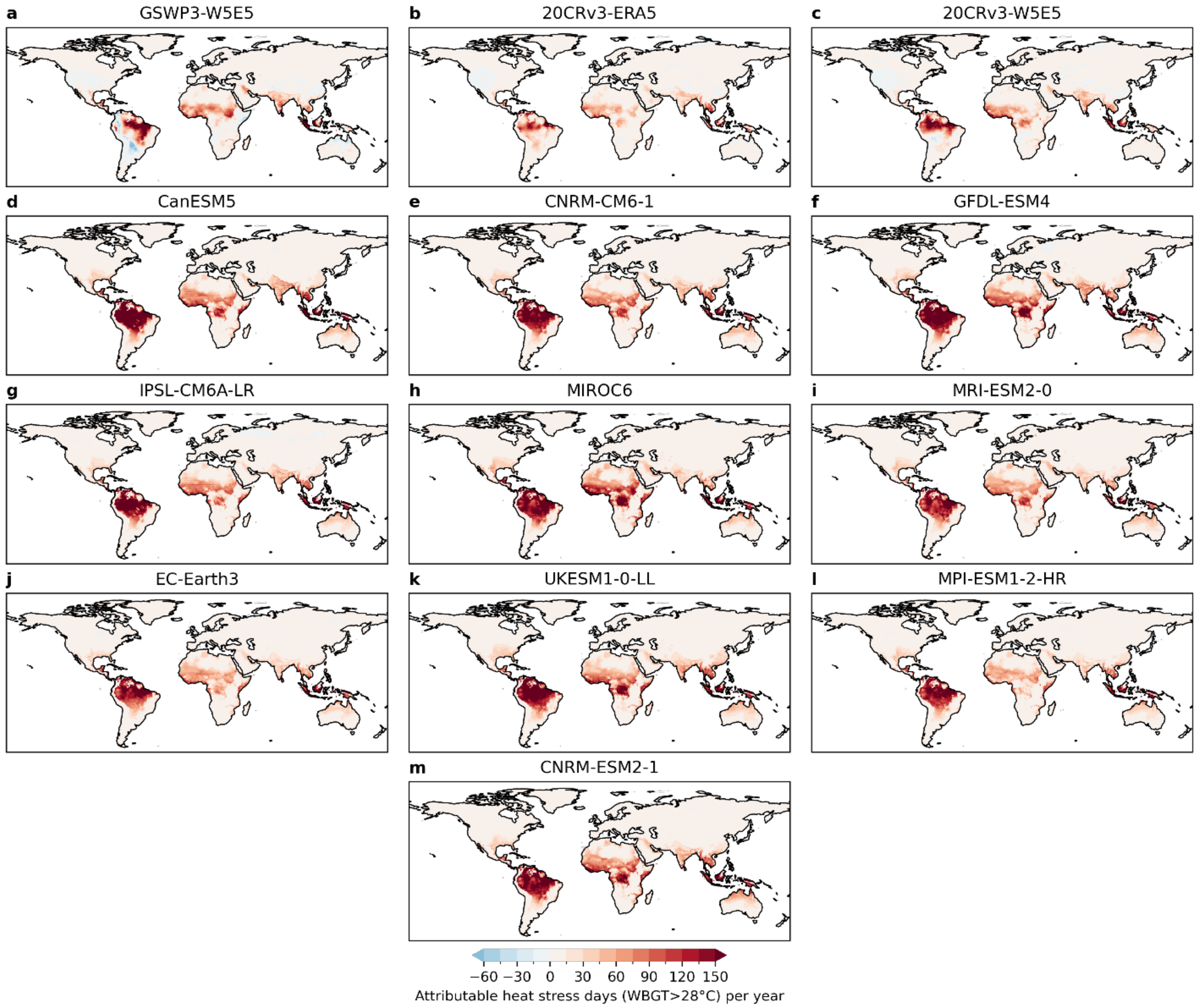

Fig. S1.

Number of attributable heat stress days per year (WBGT>28ºC) from reanalysis (a-c) and climate model simulations (d-m) at 2023 warming levels. In (a-c) attributable days per year are estimated using non-stationary statistical distribution modelling, comparing present day warming levels with a pre-industrial 1.3ºC colder world (see Methods). In (d-m) attributable days are estimated using empirical percentiles on bias-adjusted and statistically downscaled historical (natural and anthropogenic forcings included) CMIP6 simulations, extended with SSP3-RCP7.0, comparing the 1850-1900 period to a 30-year period matching present day warming levels in each model (see Methods). Results over oceans are masked out.

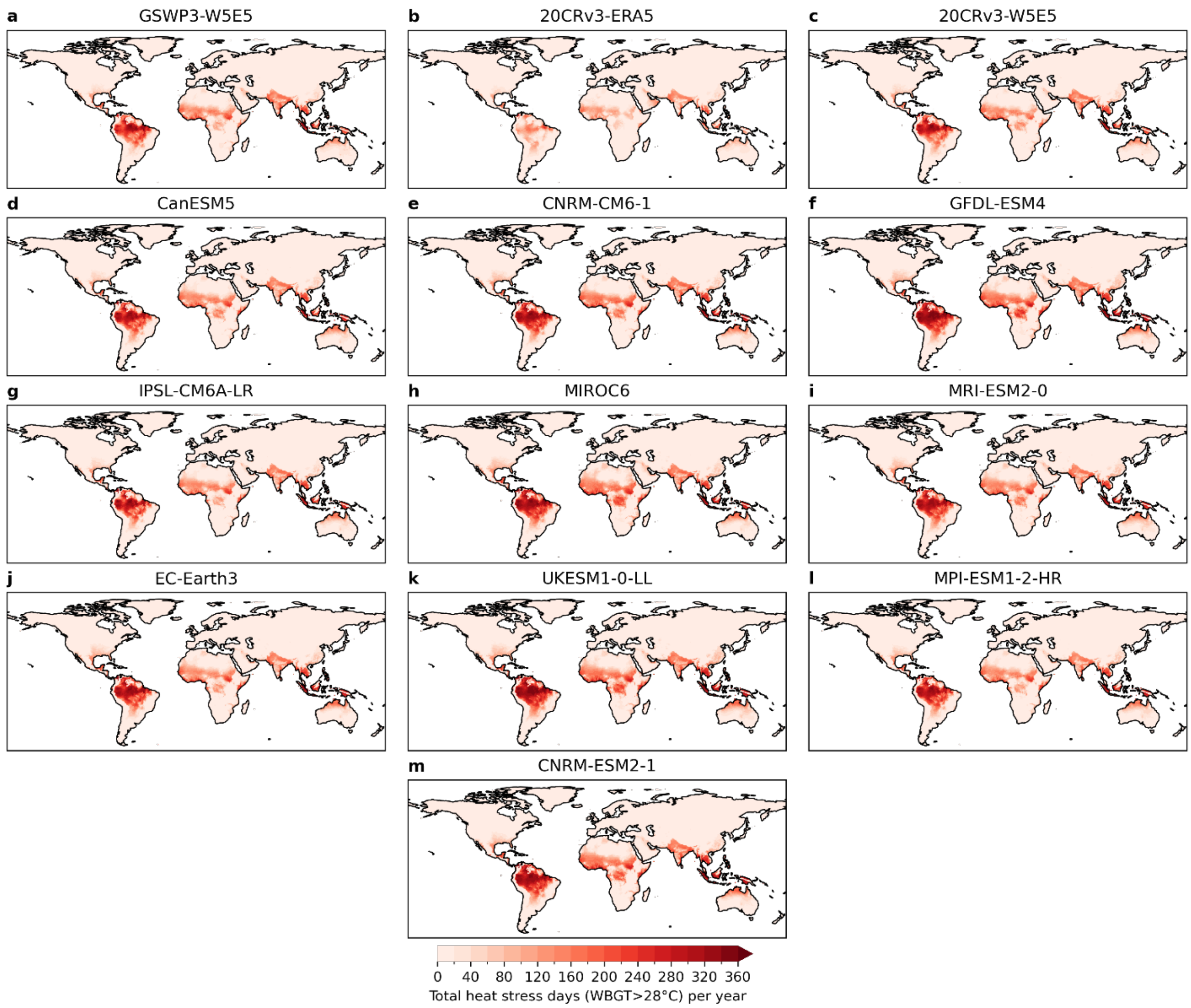

Fig. S2.

Number of total heat stress days per year (WBGT>28ºC) at present-day (2023) warming levels from reanalysis (a-c) and climate model simulations (d-m). In (a-c) total days per year are estimated using non-stationary statistical distribution modelling and calculating the expected probability of threshold exceedance at present day warming levels. In (d-m) attributable days are estimated using empirical percentiles on bias-adjusted and statistically downscaled historical and SSP3-RCP7.0 CMIP6 simulations, based on a 30-year period matching present day warming levels in each model (see Methods). Results over oceans are masked out.

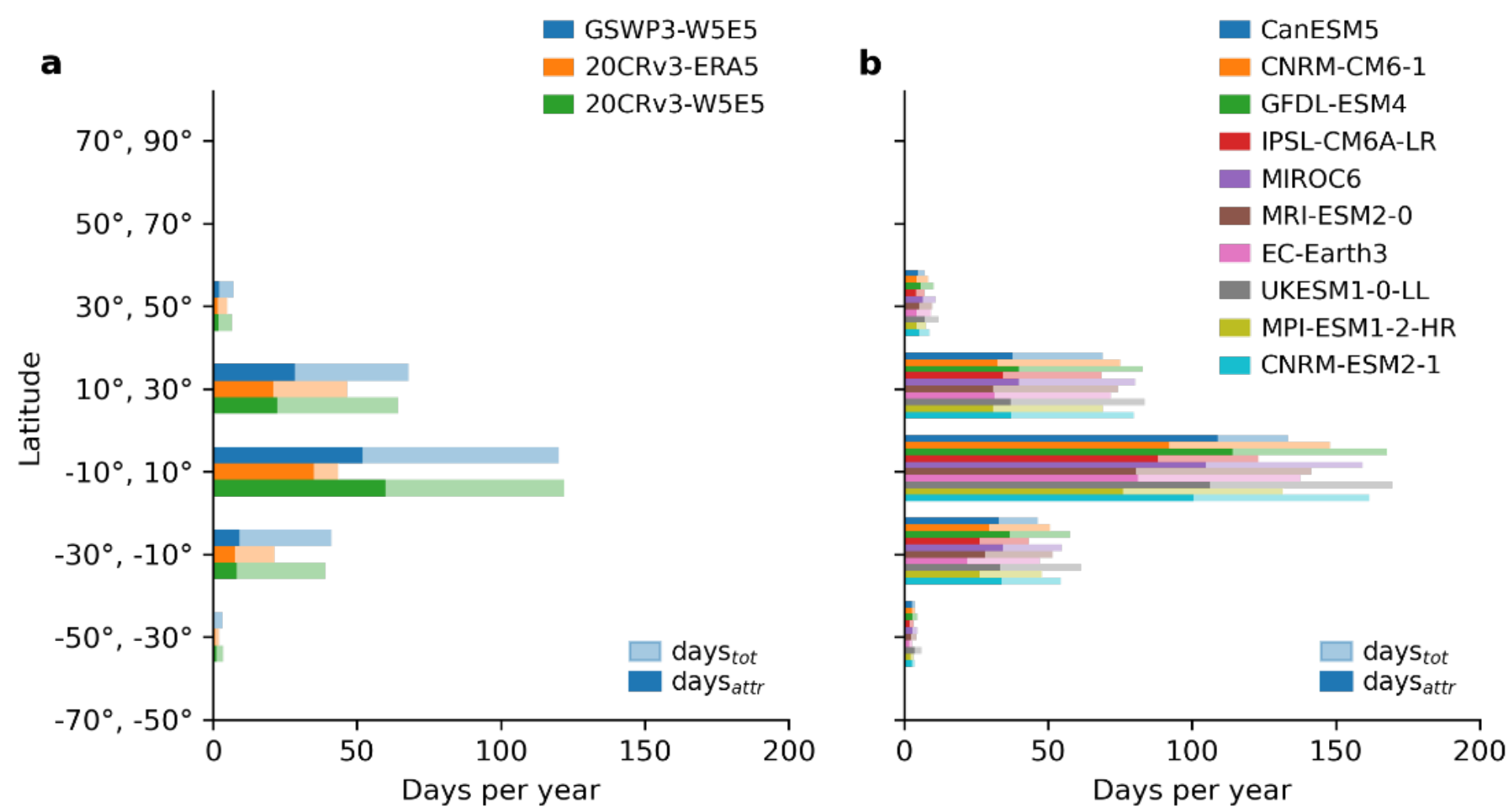

Fig. S3.

Number of total ($days_{tot}$) and attributable ($days_{attr}$) heat stress days per year (WBGT>28ºC) from reanalysis (a) and climate model simulations (b), shown as zonal land averages from 70ºS to 90ºN. In (a) total and attributable hot-humid days per year at present day warming levels are estimated from non-stationary statistical distribution modelling comparing present day warming levels to a pre-industrial 1.3ºC colder world , in (b) total and attributable days are estimated using empirical percentiles on bias-adjusted and statistically downscaled historical CMIP6 simulations, comparing the 1850-1900 period to a 30-year period matching present day warming levels in each model (See Methods). Estimates are made at the grid-scale level and aggregated to latitude bands as land area-weighted averages, best estimate for each dataset is shown.

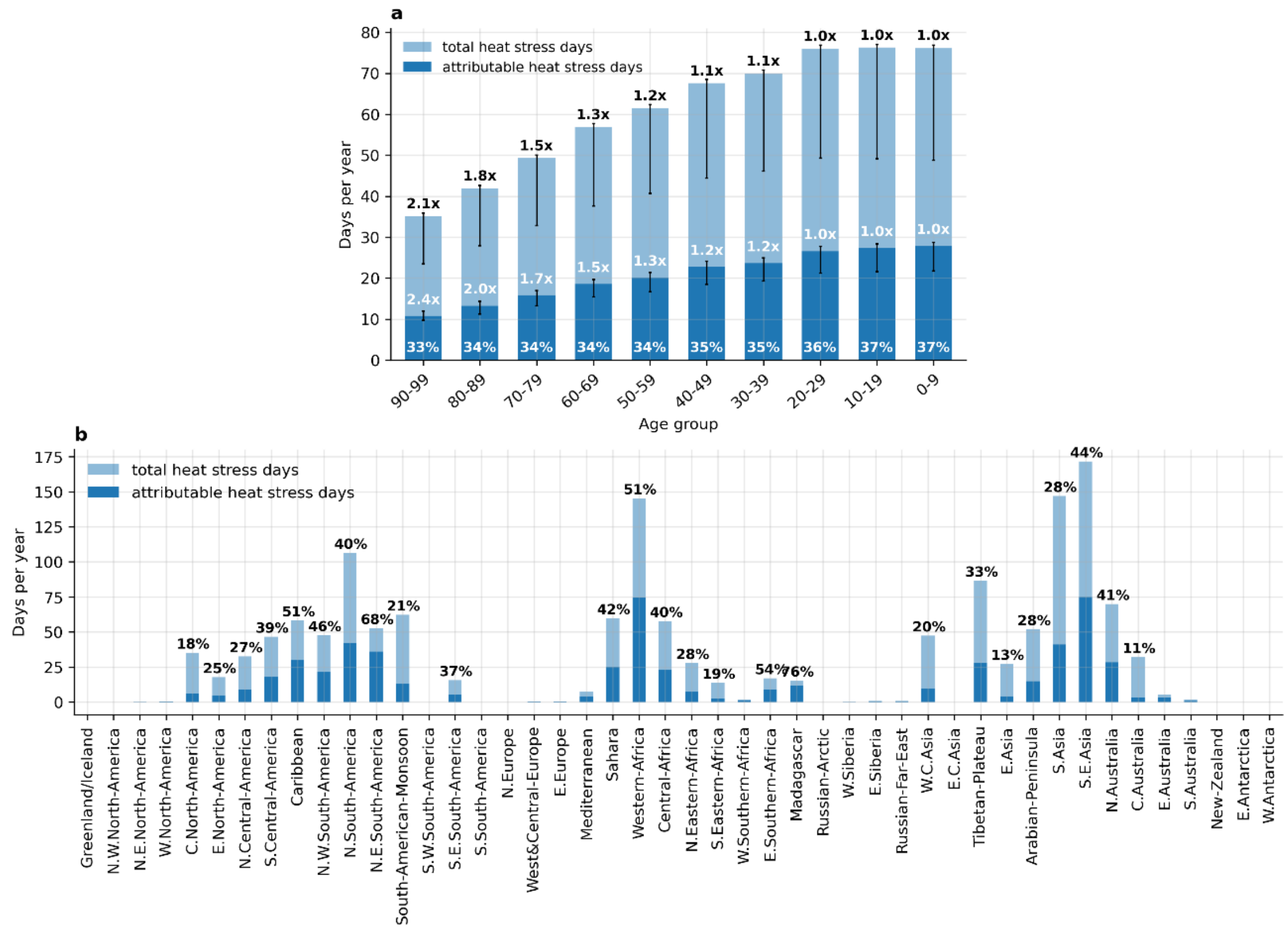

Fig S4. Comparing patterns of exposure to total and attributable heat stress, per age group and region. (a) Average number of total and attributable heat stress days experienced by each age group, globally. Multiplication factors show the relative exposure of the 0-9 age group compared to all other age groups. Percentages show percentage of total heat stress exposure that is attributable for each age group. (b) Average number of total and attributable heat stress days experienced per AR6 region. Percentages show percentage of total heat stress exposure that is attributable in each region. Bar height and error bars show median and range across reanalysis products.

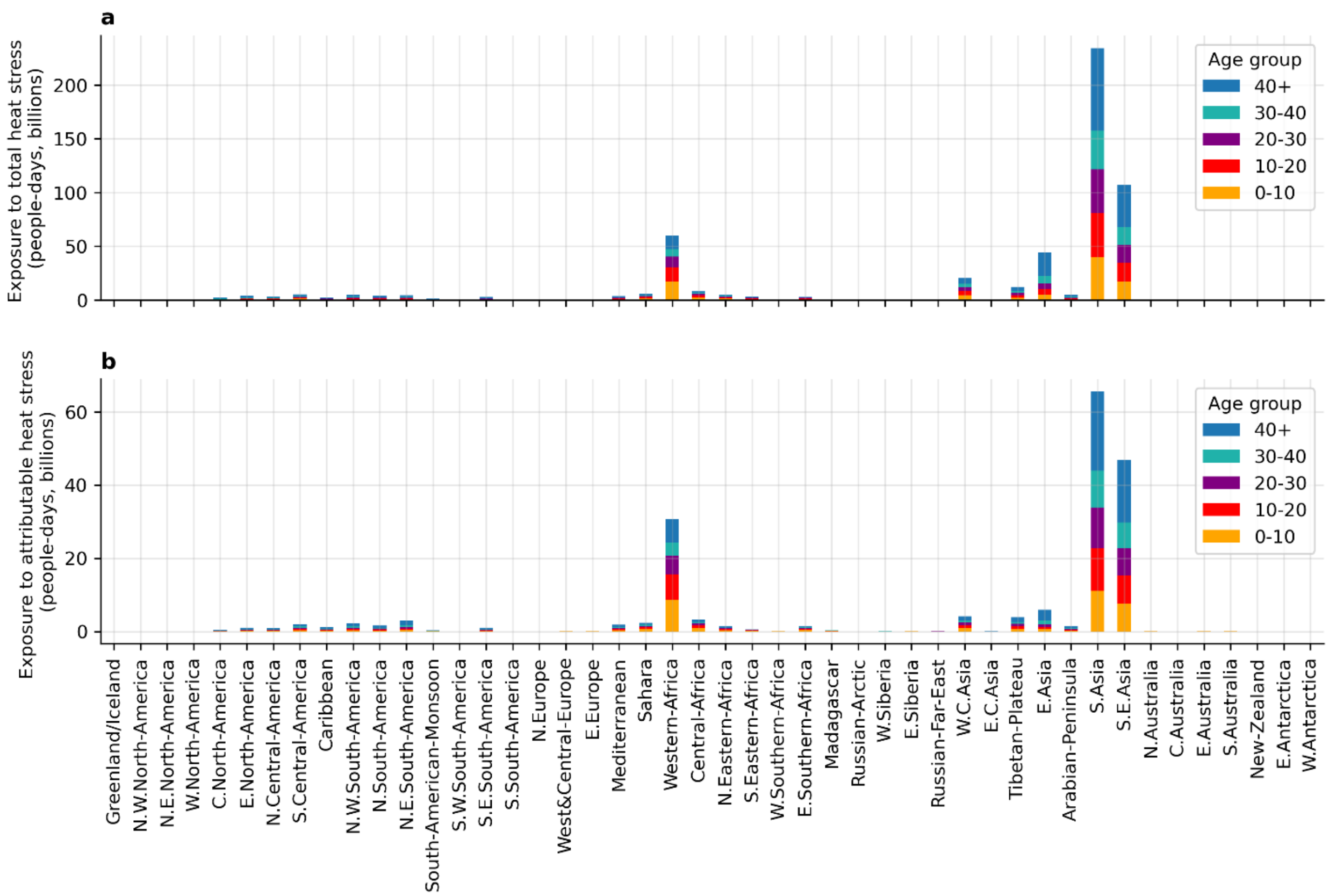

Fig S5. Population exposure to total and attributable heat stress expressed as people-days, per AR6 region and age group. Population exposure to total (c) and attributable (d) heat stress days per AR6 region, expressed in terms of people times days of exposure, and divided by the contribution of each age group. Bar height shows median across reanalysis products.

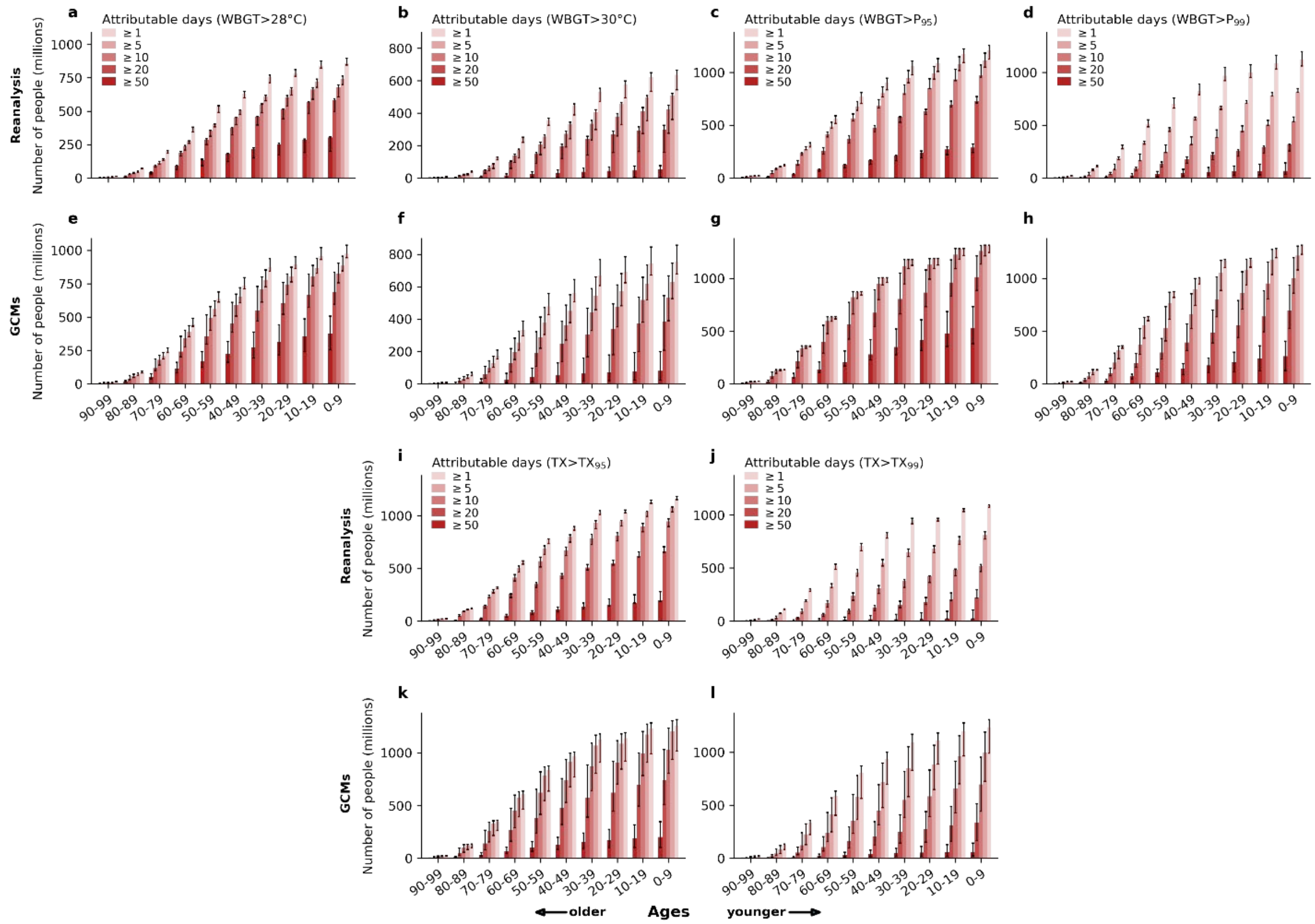

Fig. S6.

Number of people in each age group exposed to additional hot and hot-humid days under different definitions of a hot(-humid) day and frequency changes. Hot(-humid) day definitions shown: WBGT > 28 ºC, WBGT > 30 ºC, WBGT > 95th percentile in pre-industrial, WBGT > 99th percentile in pre-industrial, daily maximum temperature TX > 95th percentile in pre-industrial and daily maximum temperature TX > 99th percentile in pre-industrial. Frequency changes shown: 1, 5, 10, 20, 50 attributable days per year. Error bars show median and range across best estimates of models/datasets.

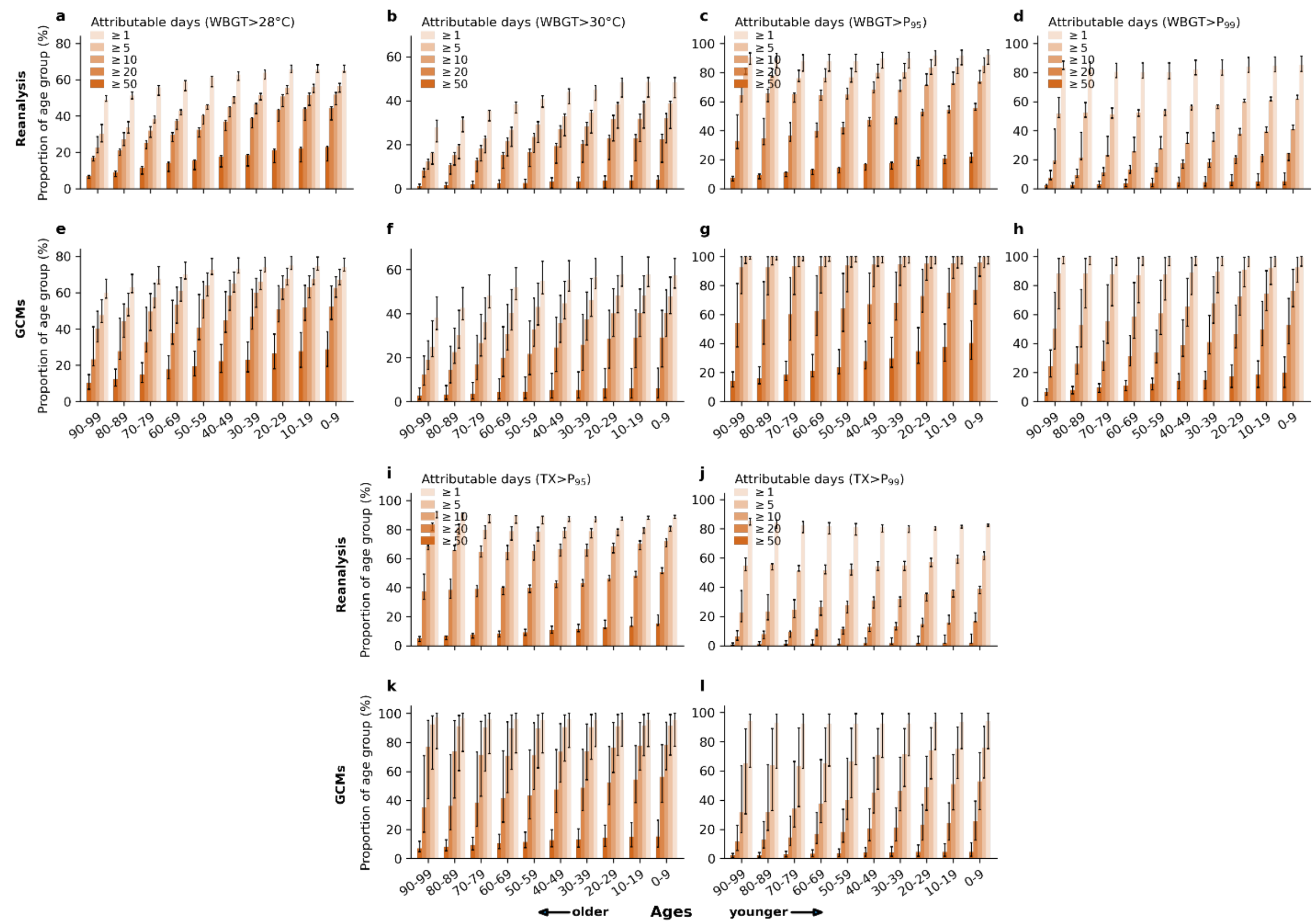

Fig. S7.

Proportion of each age group exposed to additional hot and hot-humid days under different definitions of a hot(-humid) day and frequency changes. As Fig. S6 but for proportions instead of absolute numbers. Hot(-humid) day definitions shown: WBGT > 28 ºC, WBGT > 30 ºC, WBGT > 95th percentile in pre-industrial, WBGT > 99th percentile in pre-industrial, daily maximum temperature TX > 95th percentile in pre-industrial and daily maximum temperature TX > 99th percentile in pre-industrial. Frequency changes shown: 1, 5, 10, 20, 50 attributable days per year. Error bars show median and range across best estimates of models/datasets.

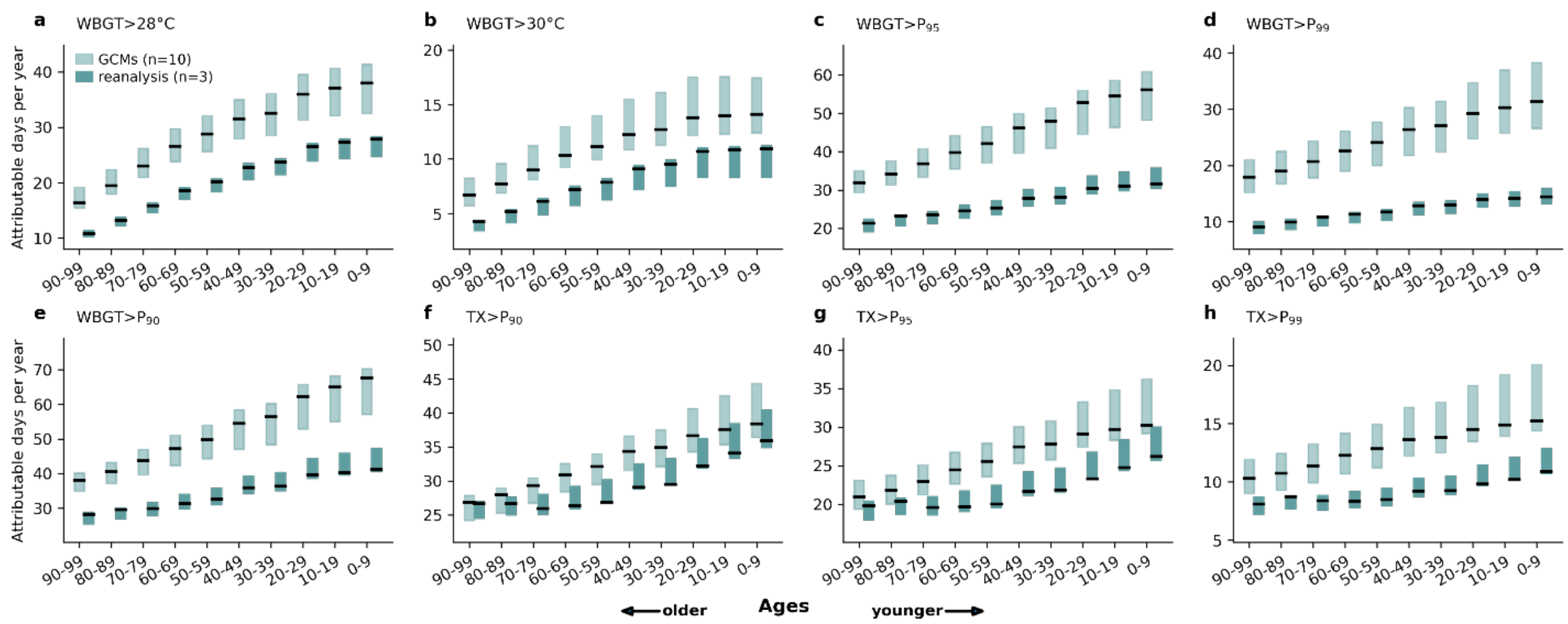

Fig. S8.

Average number of attributable days experienced per person per year, for different age groups under different definitions of a hot(-humid) day, for reanalysis and climate model simulations. Hot(-humid) day definitions shown: WBGT > 28 ºC, WBGT > 30 ºC, WBGT > 90th , 95th and 99th percentile in pre-industrial, TX > 90th , 95th 99th percentile in pre-industrial. Error bars show median and IQR across best estimates of models/datasets.

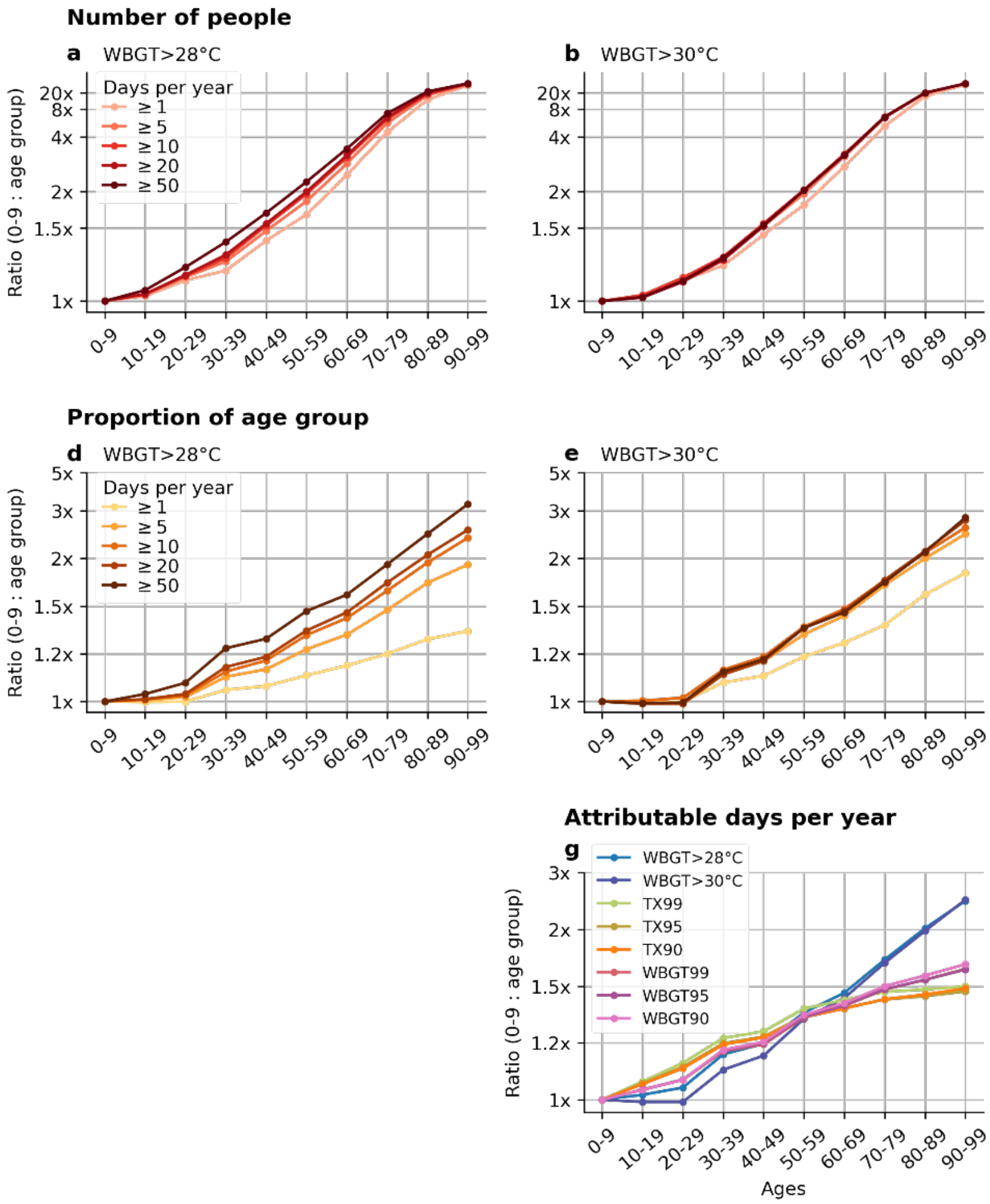

Fig. S9.

Multiplication factor (ratio) of exposure of 0-9 age group compared to other age groups, median across reanalyses. (a-c) Number of people of each age group exposed to different definitions of a (hot-)humid day and frequency changes, normalized against the 0-9 age cohort, expressed as the multiplication factor of the 0-9 cohort compared to each other age group. (d-f) As (a-c) but for the proportion of the age group. (g) As above, but for the average number of attributable days experienced by each cohort under different definitions of a (hot)-humid day. Exposure to attributable days is calculated as median from three reanalysis datasets (see Methods).

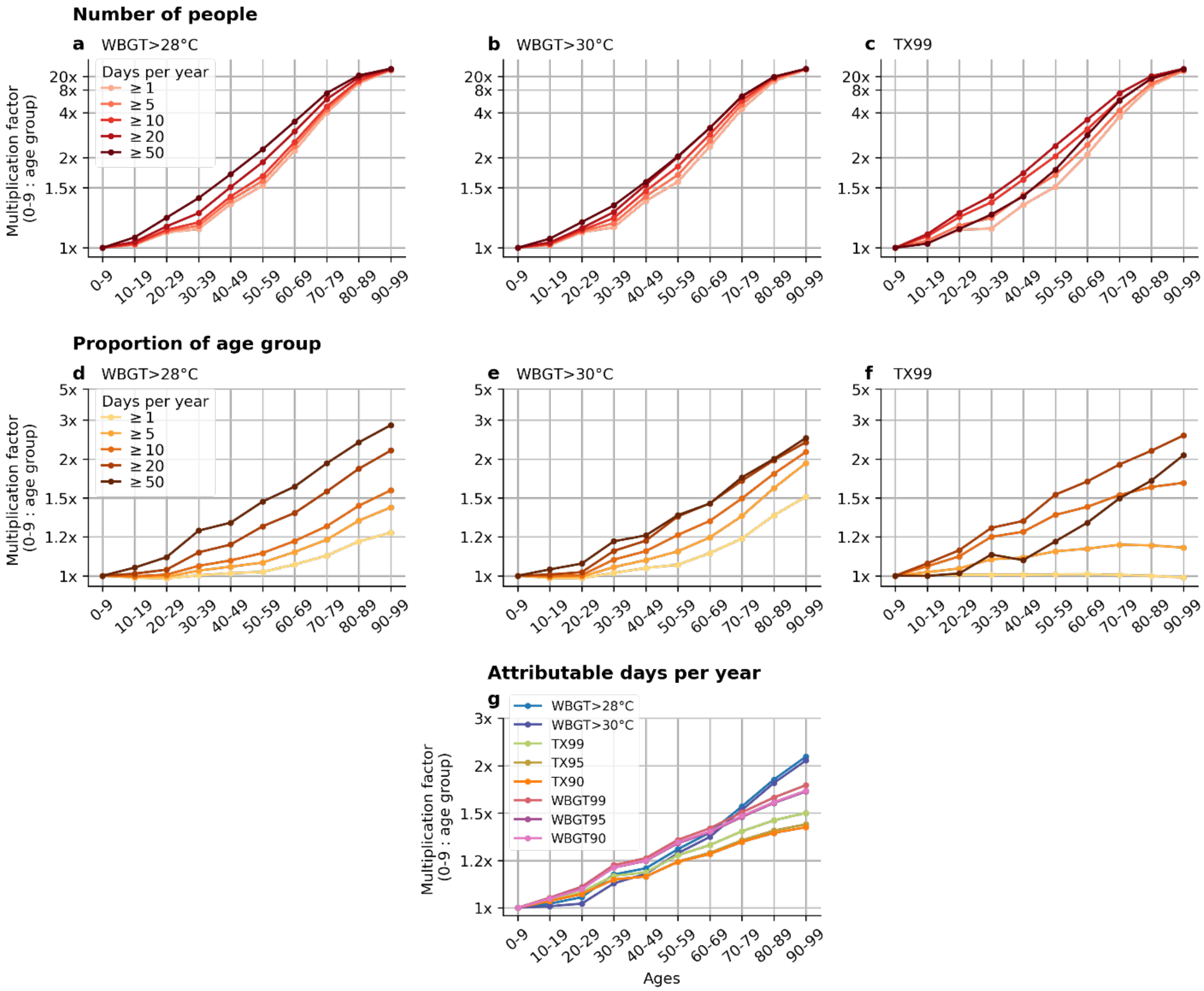

Fig. S10.

Multiplication factor (ratio) of exposure of 0-9 age group compared to other age groups, median across GCMs. As Fig. S9 but estimated from GCMs.

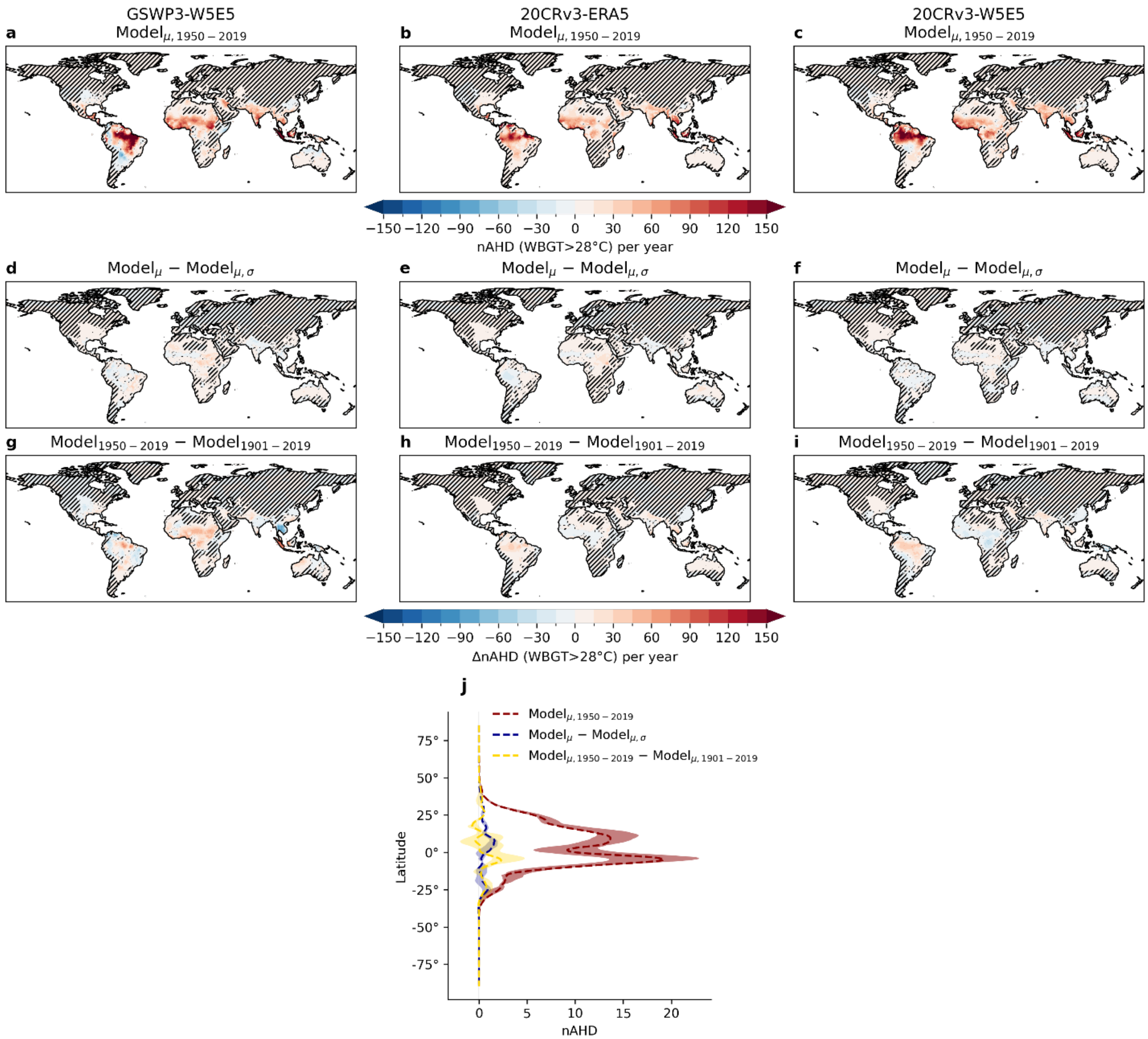

Fig. S11.

Sensitivity of shift fit attribution method on reanalysis to choice of statistical model and time-period of data, for different reanalysis datasets. (a-c) Number of attributable heat stress days per year (nAHD) with WBGT>28ºC estimated from the baseline model used in paper, i.e. through a non-stationary month-specific model where only the location parameter (μ) varies as a linear function of GMST, using data for the period 1950-2019, across 3 reanalysis datasets. (d-f) Difference between (a-c) and a model where both the location and scale parameters (μ,σ) are allowed to vary as linear functions of GMST. (g-i) Difference between (a-c) and a model where all available data from the period 1901-2019 is used. In all cases, nAHD is calculated by comparing present day warming levels with a pre-industrial 1.3ºC colder world. Hatched areas indicate locations where the absolute value of nAHD or ΔnAHD is less than 1. (j) Zonal mean of nAHD in baseline model, and of differences (ΔnAHD) when the scale parameter is allowed to vary, and when using a different time-period of data (median and range across reanalyses).

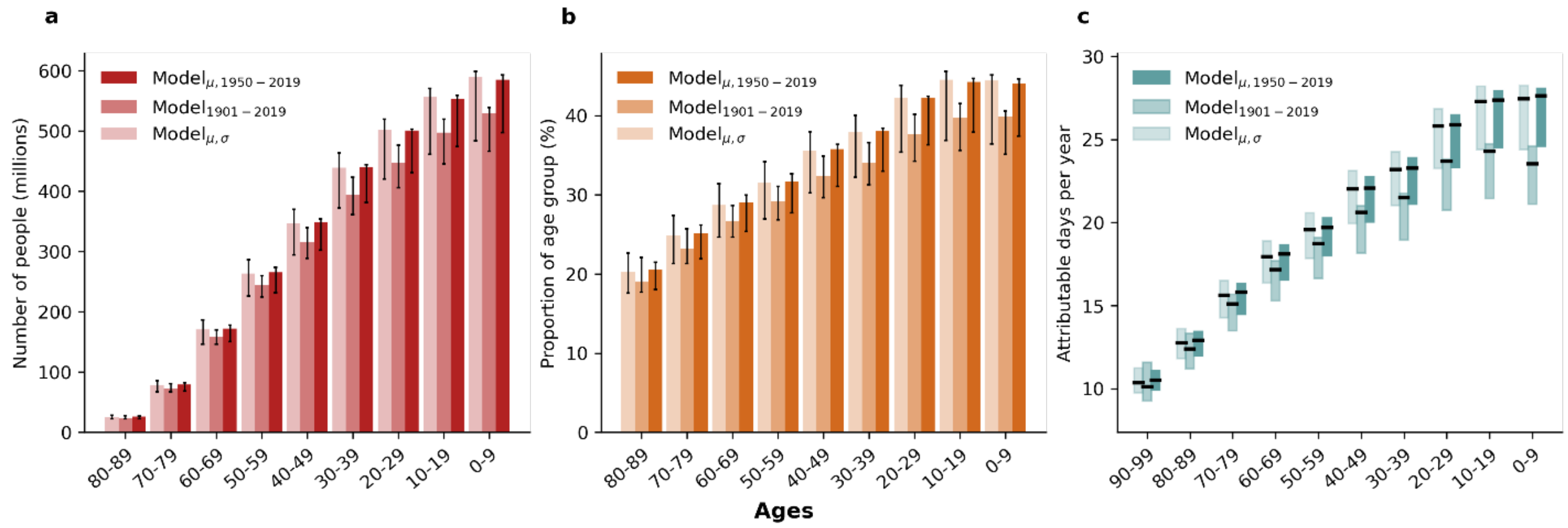

Fig. S12.

Sensitivity of exposure results to shift fit attribution method used for reanalysis. As Fig. 2 but comparing results using baseline model with parameters estimated from a different time period of data and using a model where variance is allowed to vary. (a) Number of people per age group and (b) proportion of age group exposed to 20 or more attributable heat stress days per year, (c) average (mean) number of days experienced per capita by each age group. In (a-b) multi dataset median and range are shown, in (c) multi-dataset median and IQR is shown.

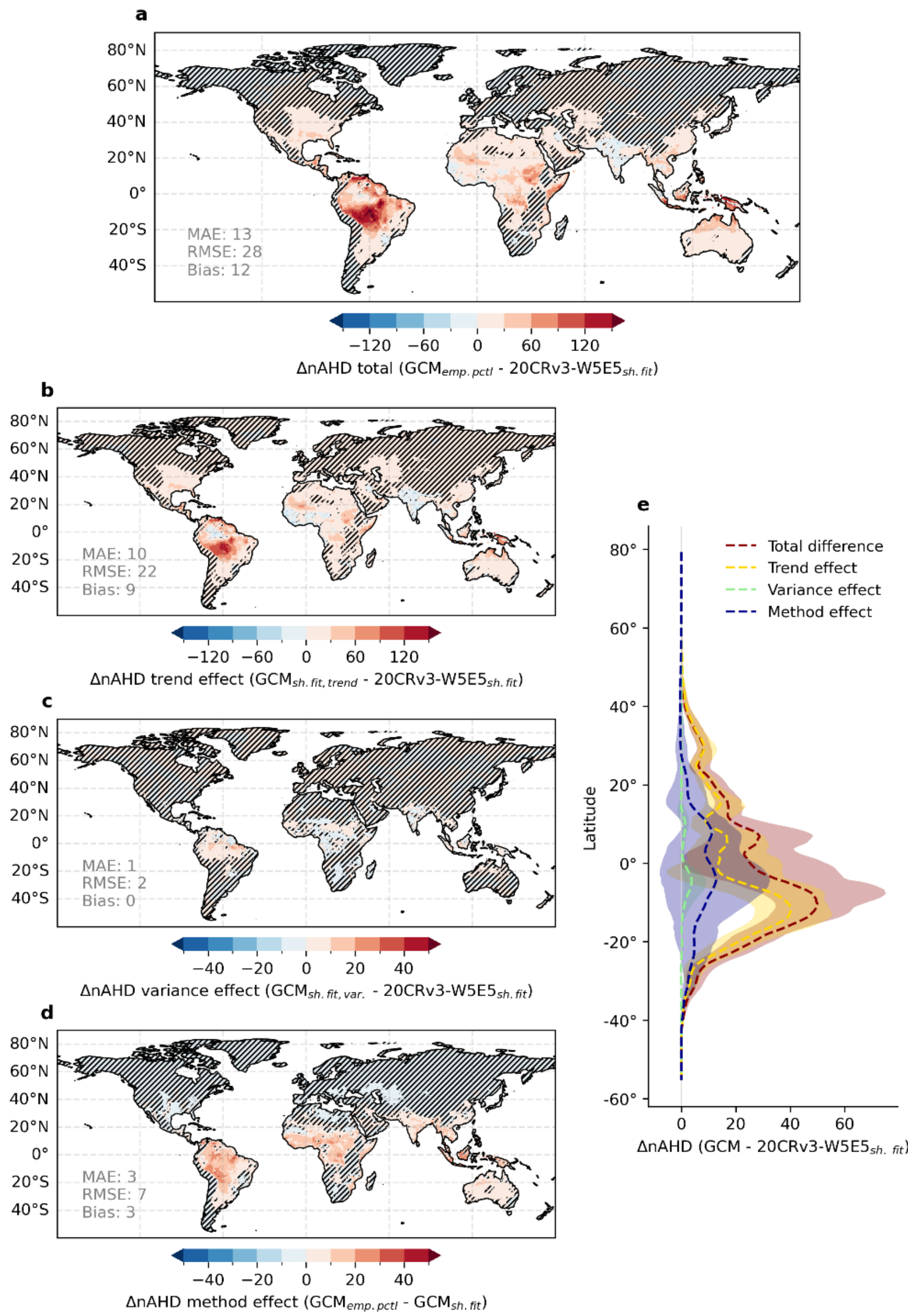

Fig S13.

Decomposing the difference in attributable heat stress days between GCMs and reanalyses. (a) Total difference in number of attributable heat stress days (nAHD, WBGT>28ºC) per year between GCMs (multi-model mean) and reanalysis (20CRv3-W5E5), and decomposition due to difference in (b) trends, (c) variance, (d) method. To isolate the trend effect, the shift fit method is applied to both reanalysis and GCMs and nAHD is calculated by holding the variance in each grid cell fixed to the estimate obtained from reanalysis. To isolate the variance effect, the trend is held fixed to estimates from reanalysis while the variance estimate is taken from GCMs. To estimate the method effect, nAHD estimates are compared using the empirical percentile and the shift fit methods on GCMs. Areas are hatched where the absolute difference is less than 1 day per year. Note different colorbars between a-b and c-d. (e) Zonal area-weighted average of each component, showing multi-model mean and range.

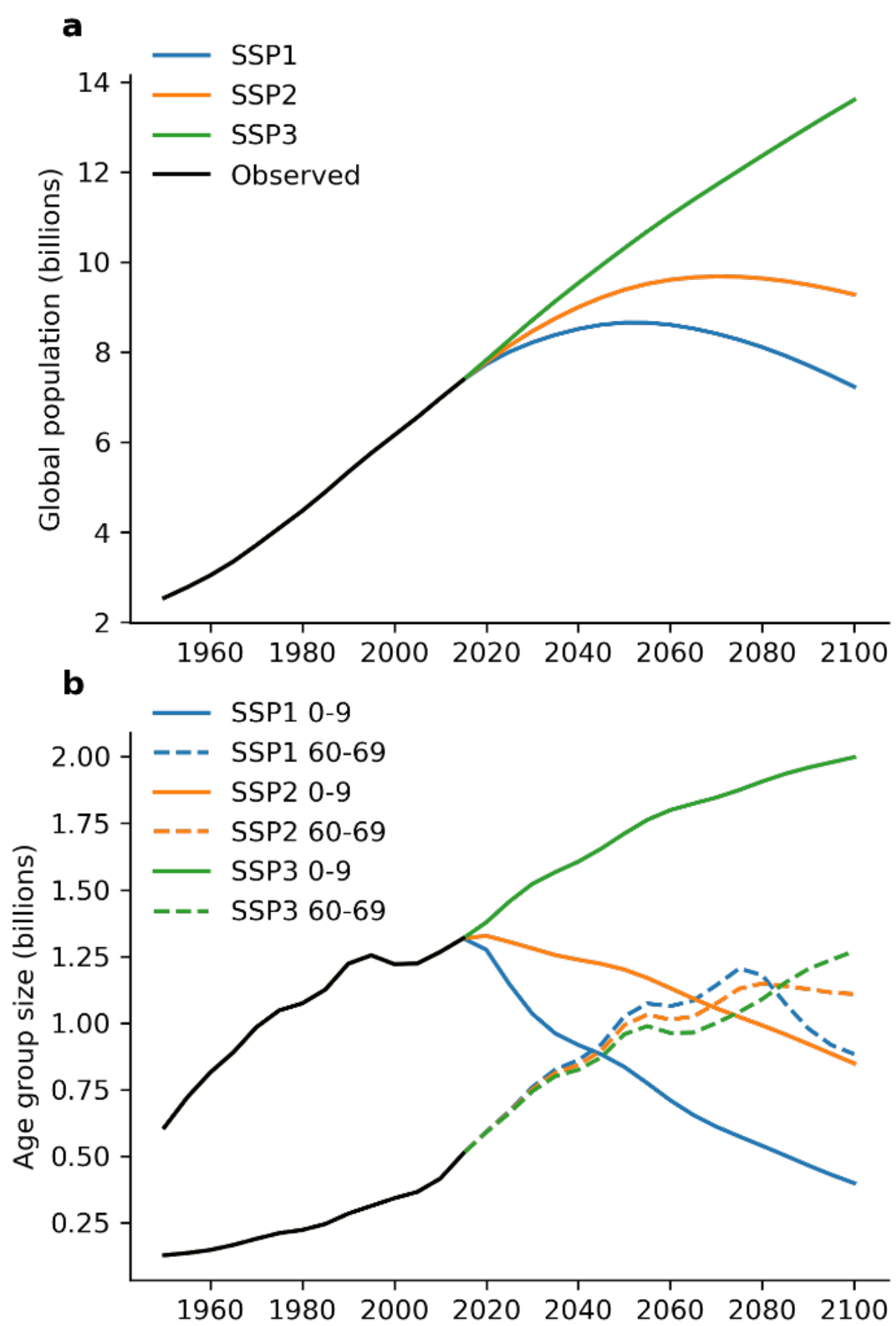

Fig. S14.

Demographic changes projected across SSPs. (a) Global population size reconstruction and projections for SSP1, SSP2 and SSP3. (b) Global size of 0-9 and 60-69 age groups, reconstructions and projections for SSP1, SSP2, SSP3. Data from WCDE v2 (see Methods).

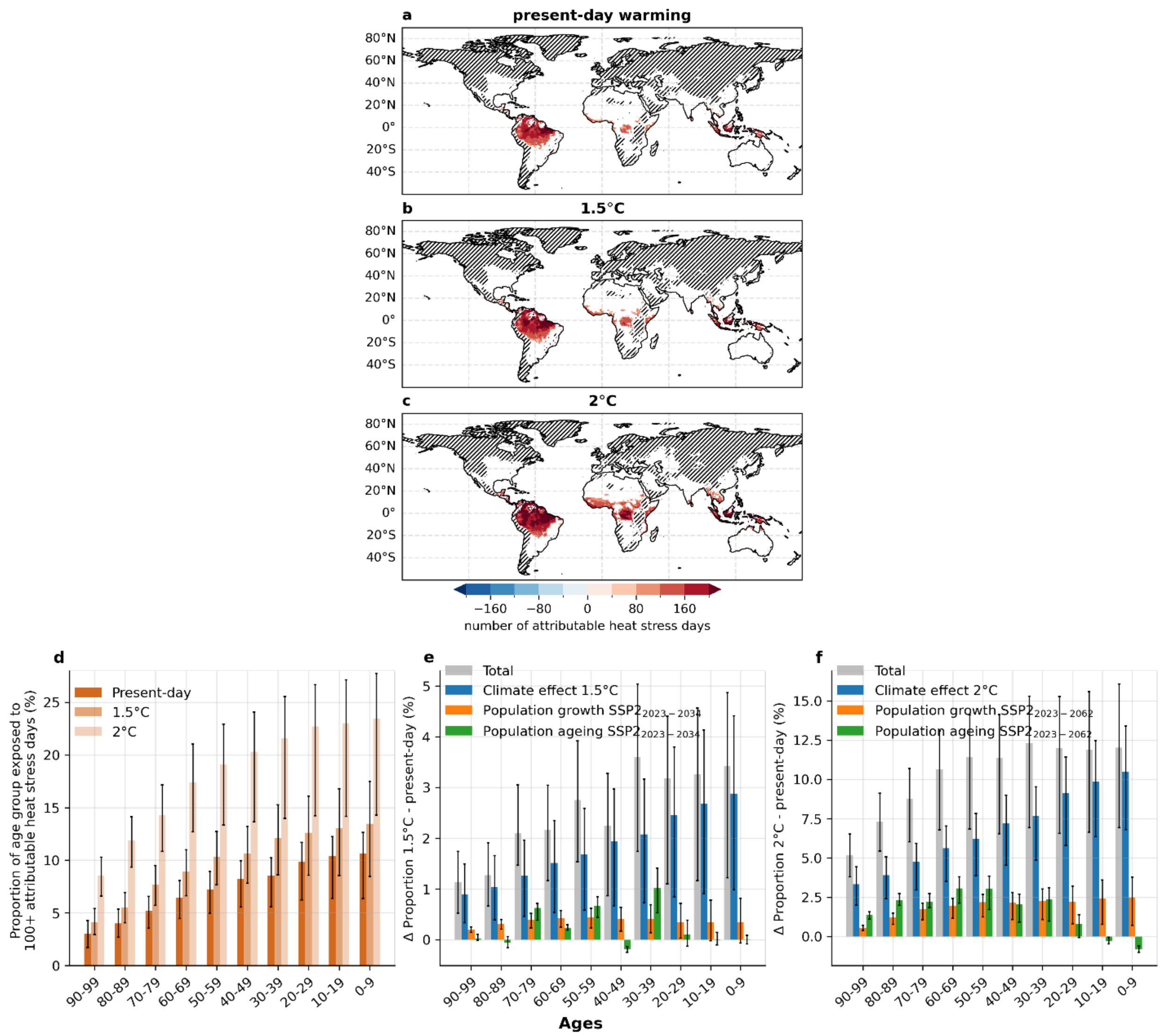

Fig. S15.

Increase in global population exposed to 100 or more attributable heat stress days per year at 1.5 and 2 of global warming. (a-c) Number of attributable heat stress days (nAHD, WBGT>28ºC) per year at present-day warming, 1.5ºC and 2ºC of global warming, estimated from GCMs. Only areas experiencing 100 or more attributable heat stress days per year are colored, and hatched areas indicate where the absolute number of attributable heat stress days is less than 1 day per year. (d) Percentage of each age group globally exposed to 100 or more attributable heat stress days under present-day warming, 1.5ºC and 2ºC. (e-f) Decomposition of the climatic and demographic drivers of changes in exposure at 1.5ºC and 2ºC, respectively. Figures show the total change in percentage exposed to 100 or more days for each age group ('Total'), the climate effect obtained by holding population data constant to 2023 levels ('Climate effect'), the population growth effect estimated by keeping age-structure constant to 2023 levels but allowing population to grow according to SSP2 ('Population growth'), and the population ageing effect estimated by additionally allowing age-structures to change according to SSP2 ('Population ageing').

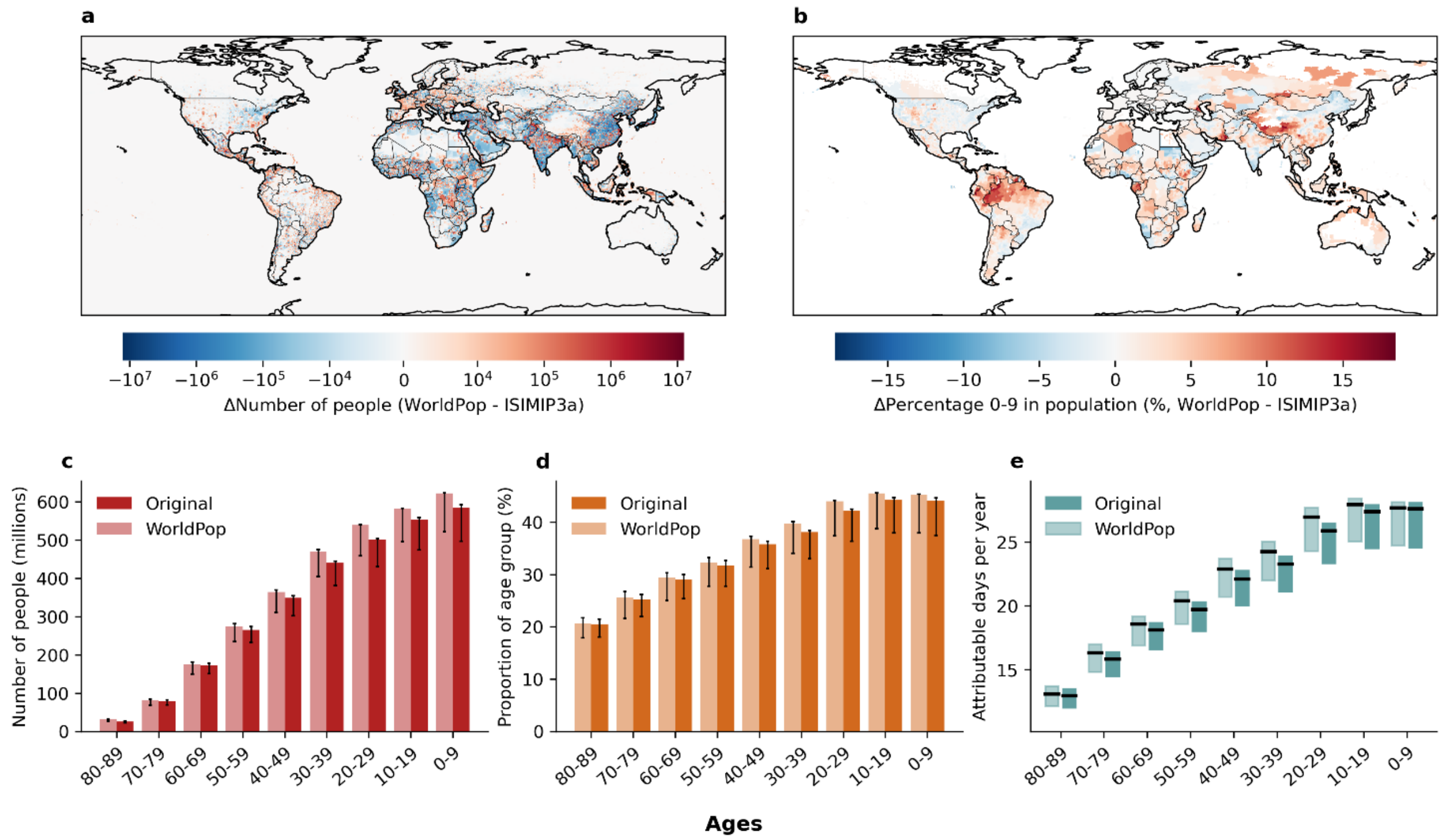

Fig. S16.

Sensitivity to gridded demographic data and the assumption of constant age-group distribution within each country. (a) Difference in number of people per grid cell between WorldPop and ISIMIP3a data, for the year 2020. (b) Difference in the percentage of people in each grid cell aged 0-9 between WorldPop and ISIMIP3a using our original method of age group disaggregation assuming constant within-country age group distribution, for the year 2020. (c-d) As Fig. 2 but comparing the effect of using our original method of age group disaggregation applied on ISIMIP3 data with WorldPop gridded age-structure data. For both approaches, we use demographic data for the year 2020 and calculate attributable heat stress days for present day warming levels from reanalysis products. (c) Total number of people of each age group exposed to at least 20 attributable heat stress days (WBGT > 28 ºC) per year at present-day warming levels, (d) Proportion of age group exposed to at least 20 attributable heat stress days (WBGT > 28 ºC) per year at present-day warming levels, (e) average number of attributable heat stress days (WBGT > 28 ºC) experienced per year by an average member of each age group at present-day warming levels. Error bars show median and range across reanalysis datasets in (c,d) and median and inter-quartile range (IQR) across datasets/models in (e).

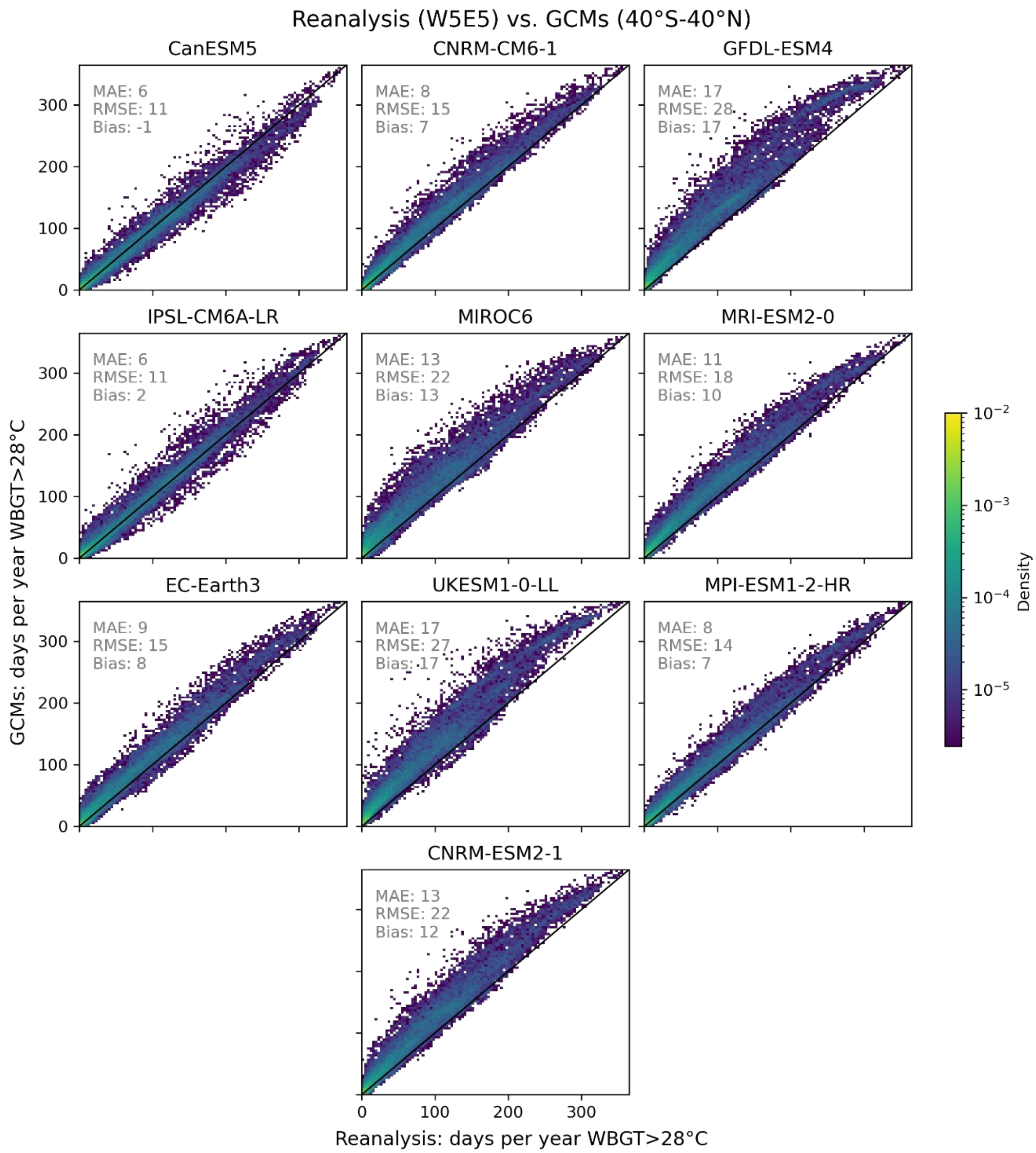

Fig. S17.

Comparing the return period of the WBGT 28ºC threshold in models and reanalysis for matching warming levels. Number of days per year crossing the WBGT 28ºC threshold in reanalysis W5E5 (x-axis) and bias-adjusted climate models (y-axis). Reanalysis years 2000-2019 are used, compared to a 20-year period in climate models with a matching warming level. Only land grid cells located 40ºS-40ºN are plotted, to exclude areas where the heat stress threshold is very rarely crossed at present-day warming levels. Evaluation metrics shown are area-weighted mean absolute error (MAE), root mean squared error (RMSE) and mean bias, computed for the number of days crossing the threshold over land grid cells located 40ºS-40ºN.

Table S1.

Central year of 30-year period used in analysis of climate models to represent present day (2023) warming level, a 1.5ºC and 2 ºC warmer world. For 2023, we match models and observations based on the end of a 10-year trend of GMST (taking observed warming from Forster et al. 2024). For 1.5ºC and 2ºC warming levels, we find the central year of a 30-year period in which average GMST crosses the respective warming level compared to 1850-1900. In both cases a 30-year period centered on the indicated year is used in analyses. Model simulations are historical and SSP3-RCP7.0, bias-adjusted and downscaled with ISIMIP3BASD. See Methods.

**Model**	**2023 warming level**	**1.5ºC**	**2 ºC**
CanESM5	2009	2012	2024
CNRM-CM6-1	2025	2032	2046
CNRM-ESM2-1	2030	2037	2052
EC-Earth3	2017	2023	2039
GFDL-ESM4	2035	2043	2058
IPSL-CM6A-LR	2012	2020	2035
MIROC6	2038	2044	2060
MPI-ESM1-2-HR	2024	2035	2052
MRI-ESM2-0	2024	2031	2046
UKESM1-0-LL	2022	2023	2032

Table S2.

Demographic disproportionality under present-day, 1.5ºC and 2ºC warming from GCMs and sensitivity to demographic changes. Columns 1-3: baseline (present-day warming + SSP2 2023 demographics, 1.5ºC and 2ºC warming + SSP2-RCP45 demographics). Columns 4-5: demographics from SSP3-RCP45 instead of SSP2-RCP45. Columns 6-7: present-day demographics held fixed. Columns 8-9: demographics from SSP2 and SSP3 under RCP60 instead of RCP45. For a given warming level, the climate conditions do not change between columns, but only demographics change, depending on the SSP. The time period of demographic data chosen is shown as year in brackets and is based on when a marker SSP-RCP scenario crosses the given warming level (see Methods). Multi-model median is shown.

	**Number of attributable days per year**	**Present-day warming + SSP2 (2023)**	**1.5°C + SSP2-45 (2034)**[1]	**2°C + SSP2-45 (2062)**[2]	**1.5°C + SSP3-45 (2033)**[3]	**2°C + SSP3-45 (2062)**[4]	**1.5°C + SSP2 (2023)**	**2°C + SSP2 (2023)**	**2°C + SSP2-60 (2056)**	**2°C + SSP3-60 (2051)**
**No. people (0-9) total** billions		1.3	1.3	1.1	1.5	1.8	1.3	1.3	1.2	1.7
**People exposed (0-9)** % (millions)	1+	74% (970)	75% (940)	78% (870)	75% (1160)	78% (1410)	75% (990)	79% (1040)	78% (910)	78% (1350)
	50+	29% (380)	34% (430)	45% (500)	34% (530)	46% (820)	33% (430)	43% (560)	45% (520)	45% (780)
	100+	11% (140)	13% (170)	23% (260)	13% (210)	24% (430)	13% (170)	22% (280)	23% (270)	23% (400)
	150+	3% (40)	4% (50)	10% (120)	4% (60)	10% (190)	4% (60)	10% (140)	10% (120)	10% (180)
**No. people (60-69) total** billions		0.6	0.8	1	0.8	1	0.6	0.6	1	1
**People exposed (60-69)** % (millions)	1+	70% (450)	75% (600)	80% (820)	74% (580)	80% (770)	73% (460)	77% (490)	80% (820)	80% (770)
	50+	18% (110)	23% (190)	39% (390)	23% (180)	39% (380)	22% (140)	30% (190)	36% (370)	35% (340)
	100+	6% (40)	9% (70)	17% (180)	9% (70)	17% (170)	8% (50)	13% (80)	16% (160)	15% (150)
	150+	3% (20)	4% (30)	9% (90)	4% (30)	9% (90)	4% (20)	7% (40)	8% (90)	8% (80)
**Ratio no. people exposed** (0-9 : 60-69)	1+	2.2	1.6	1.1	2	1.8	2.1	2.1	1.1	1.8
	50+	3.3	2.4	1.3	3	2.2	3.2	3	1.4	2.3
	100+	3.1	2.3	1.4	3	2.5	3.2	3.5	1.6	2.7
	150+	2.3	1.7	1.2	2.1	2.1	2.5	3.1	1.4	2.3
**Ratio proportion of age group exposed** (0-9 : 60-69)	1+	1.1	1	1	1	1	1	1	1	1
	50+	1.6	1.5	1.2	1.5	1.2	1.6	1.5	1.2	1.3
	100+	1.5	1.5	1.3	1.5	1.3	1.6	1.7	1.4	1.5
	150+	1.1	1.1	1.1	1.1	1.1	1.2	1.5	1.2	1.3
	**Age group**									
**Average attributable days per year experienced**	0-9	38	45	61	45	61	44	59	61	61
	60-69	27	34	54	33	54	32	44	52	50
**Ratio attributable days experienced** (0-9 : 60-69)		1.4	1.3	1.1	1.3	1.1	1.4	1.4	1.2	1.2

[1] 1.5°C is crossed under SSP2-45 in 2034. The same for SSP2-RCP34 and -RCP60. For SSP2-RCP26 this is 2036.
[2] 2°C is crossed under SSP2-45 in 2062. For SSP2-RCP34 this is 2078, for SSP2-RCP60 this is 2056.
[3] 1.5°C is crossed under SSP3-45 in 2033. The same for SSP3-RCP34. For SSP3-RCP60 this is 2032.
[4] 2°C is crossed under SSP3-45 in 2062. For SSP3-RCP60 this is 2051.

Table S3.

GCM simulations used in analysis, including ensemble member number and nominal native resolution rounded to the nearest 10 km. For all GCMs, historical (1850-2014) and SSP3-RCP7.0 (2015-2100) simulations were used, previously bias-adjusted and statistically downscaled to a common 0.5º resolution within ISIMIP Phase 3b.

**GCM**	**Ensemble member**	**Native resolution (km)**
CanESM5	r1i1p1f1	250
CNRM-CM6-1	r1i1p1f2	140
CNRM-ESM2-1	r1i1p1f2	140
EC-Earth3	r1i1p1f1	80
GFDL-ESM4	r1i1p1f1	100
IPSL-CM6A-LR	r1i1p1f1	160
MIROC6	r1i1p1f1	120
MPI-ESM1-2-HR	r1i1p1f1	80
MRI-ESM2-0	r1i1p1f1	100
UKESM1-0-LL	r1i1p1f2	140

REFERENCES

1. S. I. Seneviratne, X. Zhang, M. Adnan, W. Badi, C. Dereczynski, A. D. Luca, S. Ghosh, I. Iskandar, J. Kossin, S. Lewis, F. Otto, I. Pinto, M. Satoh, S. M., Vicente-Serrano, M. Wehner, B. Zhou, "Chapter 11: Weather and climate extreme events in a changing climate," in *Climate Change 2021: The Physical Science Basis. Contribution of Working Group I to the Sixth Assessment Report of the Intergovernmental Panel on Climate Change*, V. Masson-Delmotte, P. Zhai, A. Pirani, S. L. Connors, C. Pean, S. Berger, N. Caud, Y. Chen, L. Goldfarb, M. I. Gomis, M. Huang, K. Leitzell, E. Lonnoy, J. B. R. Matthews, T. K. Maycock, T. Waterfield, O. Yelekci, R. Yu, B. Zhou, Eds. (Cambridge Univ. Press, Cambridge, United Kingdom and New York, NY, USA, 2021), pp. 1513–1766.

2. France24, "Death toll surges as India heatwave leaves hospitals overwhelmed" (2023); www.france24.com/en/asia-pacific/20230619-death-toll-surges-as-india-heatwave-leaves-hospitals-overhwelmed.

3. R. H. White, S. Anderson, J. F. Booth, G. Braich, C. Draeger, C. Fei, C. D. G. Harley, S. B. Henderson, M. Jakob, C.-A. Lau, L. Mareshet Admasu, V. Narinesingh, C. Rodell, E. Roocroft, K. R. Weinberger, G. West, The unprecedented Pacific Northwest heatwave of June 2021. *Nat. Commun.* **14**, 727 (2023).

4. S. Y. Philip, S. F. Kew, G. J. Van Oldenborgh, F. S. Anslow, S. I. Seneviratne, R. Vautard, D. Coumou, K. L. Ebi, J. Arrighi, R. Singh, M. Van Aalst, C. Pereira Marghidan, M. Wehner, W. Yang, S. Li, D. L. Schumacher, M. Hauser, R. Bonnet, L. N. Luu, F. Lehner, N. Gillett, J. S. Tradowsky, G. A. Vecchi, C. Rodell, R. B. Stull, R. Howard, F. E. L. Otto, Rapid attribution analysis of the extraordinary heat wave on the Pacific coast of the US and Canada in June 2021. *Earth Syst. Dynam.* **13**, 1689–1713 (2022).

5. M. Zachariah, R. Vautard, R. Chandrasekaran, S. Chaithra, J. Kimutai, T. Arulalan, K. AchutaRao, C. Barnes, R. Singh, M. Vahlberg, J. Arrgihi, E. Raju, U. Sharma, A. Ogra, C. Vaddhanaphuti, C. Bahinipati, P. Tschakert, C. Pereira Marghidan, A. Mondal, C. Schwingshackl, S. Philip, F. Otto, "Extreme humid heat in South Asia in April 2023, largely driven by climate change, detrimental to vulnerable and disadvantaged communities" (Imperial College London, 2023); https://doi.org/10.25561/104092.

6. A. M. Vicedo-Cabrera, N. Scovronick, F. Sera, D. Royé, R. Schneider, A. Tobias, C. Astrom, Y. Guo, Y. Honda, D. M. Hondula, R. Abrutzky, S. Tong, M. D. S. Z. S. Coelho, P. H. N. Saldiva, E. Lavigne, P. M. Correa, N. V. Ortega, H. Kan, S. Osorio, J. Kyselý, A. Urban, H. Orru, E. Indermitte, J. J. K. Jaakkola, N. Ryti, M. Pascal, A. Schneider, K. Katsouyanni, E. Samoli, F. Mayvaneh, A. Entezari, P. Goodman, A. Zeka, P. Michelozzi, F. de'Donato, M. Hashizume, B. Alahmad, M. H. Diaz, C. D. L. C. Valencia, A. Overcenco, D. Houthuijs, C. Ameling, S. Rao, F. Di Ruscio, G. Carrasco-Escobar, X. Seposo, S. Silva, J. Madureira, I. H. Holobaca, S. Fratianni, F. Acquaotta, H. Kim, W. Lee, C. Iniguez, B. Forsberg, M. S. Ragettli, Y. L. L. Guo, B. Y. Chen, S. Li, B. Armstrong, A. Aleman, A. Zanobetti, J. Schwartz, T. N. Dang, D. V. Dung, N. Gillett, A. Haines, M. Mengel, V. Huber, A. Gasparrini, The burden of heat-related mortality attributable to recent human-induced climate change. *Nat. Clim. Chang.* **11**, 492–500 (2021).

7. A. M. Vicedo-Cabrera, E. De Schrijver, D. L. Schumacher, M. S. Ragettli, E. M. Fischer, S. I. Seneviratne, The footprint of human-induced climate change on heat-related deaths in the summer of 2022 in Switzerland. *Environ. Res. Lett.* **18**, 074037 (2023).

8. G. Cissé, R. McLeman, H. Adams, P. Aldunce, K. Bowen, D. Campbell-Lendrum, S. Clayton, K. L. Ebi, J. Hess, C. Huang, Q. Liu, G. McGregor, J. Semenza, M. C. Tirado, "Chapter 7: Health, wellbeing and the changing structure of communities," in *Climate Change 2022: Impacts, Adaptation and Vulnerability. Contribution of Working Group II to the Sixth Assessment Report of the Intergovernmental Panel on Climate Change*, H.-O. Pörtner, D. C. Roberts, M. M. B. Tignor, E. S. Poloczanska, K. Mintenbeck, A. Alegría, M. Craig, S. Langsdorf, S. Löschke, V. Möller, A. Okem, B. Rama, Eds. (Cambridge Univ. Press, 2022).

9. G. R. McGregor, J. K. Vanos, Heat: A primer for public health researchers. *Public Health* **161**, 138–146 (2018).

10. K. L. Ebi, A. Capon, P. Berry, C. Broderick, R. De Dear, G. Havenith, Y. Honda, R. S. Kovats, W. Ma, A. Malik, N. B. Morris, L. Nybo, S. I. Seneviratne, J. Vanos, O. Jay, Hot weather and heat extremes: Health risks. *Lancet* **398**, 698–708 (2021).

11. J. R. Buzan, M. Huber, Moist heat stress on a hotter Earth. *Annu. Rev. Earth Planet. Sci.* **48**, 623–655 (2020).

12. A. Dimitrova, A. Dimitrova, M. Mengel, A. Gasparrini, H. Lotze-Campen, S. Gabrysch, Temperature-related neonatal deaths attributable to climate change in 29 low- and middle-income countries. *Nat. Commun.* **15**, 5504 (2024).

13. C. J. Smith, Pediatric thermoregulation: Considerations in the face of global climate change. *Nutrients* **11**, 2010 (2019).

14. K. Knowlton, M. Rotkin-Ellman, G. King, H. G. Margolis, D. Smith, G. Solomon, R. Trent, P. English, The 2006 California heat wave: Impacts on hospitalizations and emergency department visits. *Environ. Health Perspect.* **117**, 61–67 (2009).

15. P. E. Sheffield, M. T. Herrera, E. J. Kinnee, J. E. Clougherty, Not so little differences: Variation in hot weather risk to young children in New York City. *Public Health* **161**, 119–126 (2018).

16. L. H. Schapiro, M. A. McShane, H. K. Marwah, M. E. Callaghan, M. L. Neudecker, Impact of extreme heat and heatwaves on children's health: A scoping review. *J. Clim. Change Health* **19**, 100335 (2024).

17. Z. Xu, P. E. Sheffield, H. Su, X. Wang, Y. Bi, S. Tong, The impact of heat waves on children's health: A systematic review. *Int. J. Biometeorol.* **58**, 239–247 (2014).

18. C. Y. Wright, M. Norval, N. Naidoo, M. Bulani, A. Coovadia, L. Theron, The impact of rising ambient temperatures on the mental and physical health of children. *Hyg. Environ. Health Adv.* **15**, 100137 (2025).

19. F. Perera, K. Nadeau, Climate change, fossil-fuel pollution, and children's health. *N. Engl. J. Med.* **386**, 2303–2314 (2022).

20. Z. Xu, P. E. Sheffield, W. Hu, H. Su, W. Yu, X. Qi, S. Tong, Climate change and children's health—A call for research on what works to protect children. *Int. J. Environ. Res. Public Health* **9**, 3298–3316 (2012).

21. D. Helldén, C. Andersson, M. Nilsson, K. L. Ebi, P. Friberg, T. Alfvén, Climate change and child health: A scoping review and an expanded conceptual framework. *Lancet Planet. Heath* **5**, e164–e175 (2021).

22. R. J. Park, A. P. Behrer, J. Goodman, Learning is inhibited by heat exposure, both internationally and within the United States. *Nat. Hum. Behav.* **5**, 19–27 (2021).

23. L. B. Strand, A. G. Barnett, S. Tong, The influence of season and ambient temperature on birth outcomes: A review of the epidemiological literature. *Environ. Res.* **111**, 451–462 (2011).

24. K. Grace, F. Davenport, H. Hanson, C. Funk, S. Shukla, Linking climate change and health outcomes: Examining the relationship between temperature, precipitation and birth weight in Africa. *Glob. Environ. Chang.* **35**, 125–137 (2015).

25. C. He, Y. Zhu, J. Bachwenkizi, R. Chen, H. Kan, W. W. Fawzi, Assessment of heatwave impacts on child feeding practices across 36 low-income and middle-income countries: A cross-sectional analysis. *Lancet Planet. Heath* **9**, 101281 (2025).

26. S. Blom, A. Ortiz-Bobea, J. Hoddinott, Heat exposure and child nutrition: Evidence from West Africa. *J. Environ. Econ. Manag.* **115**, 102698 (2022).

27. M. C. Thomson, L. R. Stanberry, Climate change and vectorborne diseases. *N. Engl. J. Med.* **387**, 1969–1978 (2022).

28. T. L. Symons, A. Moran, A. Balzarolo, C. Vargas, M. Robertson, J. Lubinda, A. Saddler, M. McPhail, J. Harris, J. Rozier, A. Browne, P. Amratia, A. Bertozzi-Villa, S. Bhatt, E. Cameron, N. Golding, D. L. Smith, A. M. Noor, S. F. Rumisha, M. D. Palmer, D. J. Weiss, N. Desai, D. Potere, N. Sukitsch, W. Woods, P. W. Gething, Projected impacts of climate change on malaria in Africa. *Nature* **651**, 390–396 (2026).

29. E. J. Carlton, A. P. Woster, P. DeWitt, R. S. Goldstein, K. Levy, A systematic review and meta-analysis of ambient temperature and diarrhoeal diseases. *Int. J. Epidemiol.* **45**, 117–130 (2016).

30. M. Flückiger, M. Ludwig, Temperature and risk of diarrhoea among children in Sub-Saharan Africa. *World Dev.* **160**, 106070 (2022).

31. L. Liu, H. L. Johnson, S. Cousens, J. Perin, S. Scott, J. E. Lawn, I. Rudan, H. Campbell, R. Cibulskis, M. Li, C. Mathers, R. E. Black, Global, regional, and national causes of child mortality: An updated systematic analysis for 2010 with time trends since 2000. *Lancet* **379**, 2151–2161 (2012).

32. S. Chapman, C. E. Birch, J. H. Marsham, C. Part, S. Hajat, M. F. Chersich, K. L. Ebi, S. Luchters, B. Nakstad, S. Kovats, Past and projected climate change impacts on heat-related child mortality in Africa. *Environ. Res. Lett.* **17**, 074028 (2022).

33. A. J. Wilson, R. D. Bressler, C. Ivanovich, C. Tuholske, C. Raymond, R. M. Horton, A. Sobel, P. Kinney, T. Cavazos, J. G. Shrader, Heat disproportionately kills young people: Evidence from wet-bulb temperature in Mexico. *Sci. Adv.* **10**, eadq3367 (2024).

34. M. Odhiambo Sewe, A. Bunker, V. Ingole, T. Egondi, D. Oudin Åström, D. M. Hondula, J. Rocklöv, B. Schumann, Estimated effect of temperature on years of life lost: A retrospective time-series study of low-, middle-, and high-income regions. *Environ. Health Perspect.* **126**, 017004 (2018).

35. M. M. Engdaw, A. K. Steiner, G. C. Hegerl, A. P. Ballinger, Attribution of observed changes in extreme temperatures to anthropogenic forcing using CMIP6 models. *Weather Clim. Extrem.* **39**, 100548 (2023).

36. E. M. Fischer, R. Knutti, Anthropogenic contribution to global occurrence of heavy-precipitation and high-temperature extremes. *Nat. Clim. Change* **5**, 560–564 (2015).

37. J. Giguere, D. M. Gilford, A. J. Pershing, Attributing daily ocean temperatures to anthropogenic climate change. *Environ. Res. Climate* **3**, 035003 (2024).

38. D. M. Gilford, A. Pershing, B. H. Strauss, K. Haustein, F. E. L. Otto, A multi-method framework for global real-time climate attribution. *Adv. Stat. Clim. Meteorol. Oceanogr.* **8**, 135–154 (2022).

39. L. J. Harrington, D. J. Frame, E. M. Fischer, E. Hawkins, M. Joshi, C. D. Jones, Poorest countries experience earlier anthropogenic emergence of daily temperature extremes. *Environ. Res. Lett.* **11**, 055007 (2016).

40. T. R. Knutson, J. J. Ploshay, Detection of anthropogenic influence on a summertime heat stress index. *Clim. Change* **138**, 25–39 (2016).

41. E. Hawkins, R. Sutton, Time of emergence of climate signals. *Geophys. Res. Lett.* **39**, 2011GL050087 (2012).

42. I. Mahlstein, R. Knutti, S. Solomon, R. W. Portmann, Early onset of significant local warming in low latitude countries. *Environ. Res. Lett.* **6**, 034009 (2011).

43. C. Brimicombe, C. H. B. Lo, F. Pappenberger, C. Di Napoli, P. Maciel, T. Quintino, R. Cornforth, H. L. Cloke, Wet bulb globe temperature: Indicating extreme heat risk on a global grid. *GeoHealth* **7**, e2022GH000701 (2023).

44. B. Jacklitsch, W. J. Williams, K. Musolin, A. Coca, J.-H. Kim, N. Turner, "NIOSH criteria for a recommended standard: Occupational exposure to heat and hot environments" (US Department of Health and Human Services, Centers for Disease Control and Prevention, National Institute for Occupational Safety and Health, DHHS (NIOSH), Cincinnati, Ohio, 2016).

45. J. W. Baldwin, T. Benmarhnia, K. L. Ebi, O. Jay, N. J. Lutsko, J. K. Vanos, Humidity's role in heat-related health outcomes: A heated debate. *Environ. Health Perspect.* **131**, 055001 (2023).

46. S. Kc, W. Lutz, The human core of the shared socioeconomic pathways: Population scenarios by age, sex and level of education for all countries to 2100. *Glob. Environ. Chang.* **42**, 181–192 (2017).

47. W. Lutz, N. Stillianakis, M. Stonawski, A. Goujon, K. C. Samir, *Demographic and Human Capital Scenarios for the 21st Century: 2018 Assessment for 201 Countries* (European Commission, Joint Research Centre, LU, 2018); https://data.europa.eu/doi/10.2760/835878.

48. F. Haque, F. C. Lampe, S. Hajat, K. Stavrianaki, S. M. T. Hasan, A. S. G. Faruque, T. Ahmed, S. Jubayer, I. Kelman, Impacts of climate change on diarrhoeal disease hospitalisations: How does the global warming targets of 1.5–2°C affect Dhaka, Bangladesh? *PLoS Negl. Trop. Dis.* **18**, e0012139 (2024).

49. C. J. Carlson, T. A. Carleton, R. C. Odoulami, C. D. Molitor, C. H. Trisos, The historical fingerprint and future impact of climate change on childhood malaria in Africa. medRxiv 2023.07.16.23292713 [Preprint] (2023); https://doi.org/10.1101/2023.07.16.23292713.

50. Z. Zhu, T. Zhang, T. Benmarhnia, X. Chen, H. Wang, M. Wulayin, L. D. Knibbs, S. Yang, L. Xu, C. Huang, Q. Wang, Estimating the burden of temperature-related low birthweight attributable to anthropogenic climate change in low-income and middle-income countries: A retrospective, multicentre, epidemiological study. *Lancet Planet. Heath* **8**, e997–e1009 (2024).

51. J. Vanos, G. Guzman-Echavarria, J. W. Baldwin, C. Bongers, K. L. Ebi, O. Jay, A physiological approach for assessing human survivability and liveability to heat in a changing climate. *Nat. Commun.* **14**, 7653 (2023).

52. D. J. Vecellio, Q. Kong, W. L. Kenney, M. Huber, Greatly enhanced risk to humans as a consequence of empirically determined lower moist heat stress tolerance. *Proc. Natl. Acad. Sci. U.S.A.* **120**, e2305427120 (2023).

53. C. Raymond, T. Matthews, R. M. Horton, The emergence of heat and humidity too severe for human tolerance. *Sci. Adv.* **6**, eaaw1838 (2020).

54. S. E. Perkins-Kirkpatrick, C. H. Gregory, J. K. Vanos, J. W. Baldwin, H. Staudmyer, G. Guzman-Echavarria, O. Jay, Deadly heat stress conditions are already occurring. *Nat. Commun.* **17**, 2590 (2026).

55. W. Thiery, S. Lange, J. Rogelj, C.-F. Schleussner, L. Gudmundsson, S. I. Seneviratne, M. Andrijevic, K. Frieler, K. Emanuel, T. Geiger, D. N. Bresch, F. Zhao, S. N. Willner, M. Büchner, J. Volkholz, N. Bauer, J. Chang, P. Ciais, M. Dury, L. François, M. Grillakis, S. N. Gosling, N. Hanasaki, T. Hickler, V. Huber, A. Ito, J. Jägermeyr, N. Khabarov, A. Koutroulis,

W. Liu, W. Lutz, M. Mengel, C. Müller, S. Ostberg, C. P. O. Reyer, T. Stacke, Y. Wada, Intergenerational inequities in exposure to climate extremes. *Science* **374**, 158–160 (2021).

56. K. Thomas, R. D. Hardy, H. Lazrus, M. Mendez, B. Orlove, I. Rivera-Collazo, J. T. Roberts, M. Rockman, B. P. Warner, R. Winthrop, Explaining differential vulnerability to climate change: A social science review. *WIREs Clim. Change* **10**, e565 (2019).

57. A. U. Gamage, D. Pearson, F. Hanna, A review of climate change in South East Asian Countries and human health: Impacts, vulnerability, adaptation, and mitigation. *SE Asia J. Pub. Health* **6**, 3–10 (2017).

58. F. H. Borg, J. Greibe Andersen, C. Karekezi, G. Yonga, P. Furu, P. Kallestrup, C. Kraef, Climate change and health in urban informal settlements in low- and middle-income countries—A scoping review of health impacts and adaptation strategies. *Glob. Health Action* **14**, 1908064 (2021).

59. K. Tran, G. Azhar, R. Nair, K. Knowlton, A. Jaiswal, P. Sheffield, D. Mavalankar, J. Hess, A. Cross-Sectional, Randomized cluster sample survey of household vulnerability to extreme heat among slum dwellers in Ahmedabad, India. *Int. J. Environ. Res. Public Health* **10**, 2515–2543 (2013).

60. P. R. Brown, S. Afroz, L. Chialue, T. Chiranjeevi, S. El, C. M. Grünbühel, I. Khan, C. Pitkin, V. R. Reddy, C. H. Roth, S. Sacklokham, L. J. Williams, Constraints to the capacity of smallholder farming households to adapt to climate change in South and Southeast Asia. *Clim. Dev.* **11**, 383–400 (2019).

61. C. Z. De Lima, J. R. Buzan, F. C. Moore, U. L. C. Baldos, M. Huber, T. W. Hertel, Heat stress on agricultural workers exacerbates crop impacts of climate change. *Environ. Res. Lett.* **16**, 044020 (2021).

62. M. Nilsson, A. Sie, K. Muindi, A. Bunker, V. Ingole, K. L. Ebi, Weather, climate, and climate change research to protect human health in sub-Saharan Africa and South Asia. *Glob. Health Action* **14**, 1984014 (2021).

63. G. Hagedorn, P. Kalmus, M. Mann, S. Vicca, J. Van den Berge, J.-P. van Ypersele, D. Bourg, J. Rotmans, R. Kaaronen, S. Rahmstorf, H. Kromp-Kolb, G. Kirchengast, R. Knutti, S. I. Seneviratne, P. Thalmann, R. Cretney, A. Green, K. Anderson, M. Hedberg, D. Nilsson, A. Kuttner, K. Hayhoe, Concerns of young protesters are justified. *Science* **364**, 139–140 (2019).

64. D. Bertram, ‘For you will (still) be here tomorrow’: The many lives of intergenerational equity. *Transnatl. Environ. Law* **12**, 121–149 (2023).

65. L. Parker, J. Mestre, M. Wewerinke-singh, When the kids put climate change on trial youth-focused rights-based climate litigation around the world. *J. Hum. Rights Environ.* **13**, 64–89 (2021).

66. UNCRC, General comment No. 26 (2023) on Children’s Rights and the Environment, with a Special Focus on Climate Change. *Int. Hum. Rights Law Rev.* **12**, 322–355 (2023).

67. K. Knowlton, S. Kulkarni, G. Azhar, D. Mavalankar, A. Jaiswal, M. Connolly, A. Nori-Sarma, A. Rajiva, P. Dutta, B. Deol, L. Sanchez, R. Khosla, P. Webster, V. Toma, P. Sheffield, J. Hess, The Ahmedabad heat and climate study group, development and implementation of South Asia’s first heat-health action plan in Ahmedabad (Gujarat, India). *Int. J. Environ. Res. Public Health* **11**, 3473–3492 (2014).

68. J. J. Hess, S. Lm, K. Knowlton, S. Saha, P. Dutta, P. Ganguly, A. Tiwari, A. Jaiswal, P. Sheffield, J. Sarkar, S. C. Bhan, A. Begda, T. Shah, B. Solanki, D. Mavalankar, Building resilience to climate change: Pilot evaluation of the impact of India’s first heat action plan on all-cause mortality. *J. Environ. Public Health* **2018**, 7973519 (2018).

69. H. Johar, F. I. Abdulsalam, Y. Guo, T. Baernighausen, N. K. Jahan, J. Watterson, K. Leder, D. Gouwanda, G. R. Letchuman Ramanathan, K. K. C. Lee, N. Mohamed, T. A. Zakaria, S. Barteit, T. T. Su, Community-based heat adaptation interventions for improving heat literacy, behaviours, and health outcomes: A systematic review. *Lancet Planet. Health* **9**, 101207 (2025).

70. F. Pavanello, E. De Cian, M. Davide, M. Mistry, T. Cruz, P. Bezerra, D. Jagu, S. Renner, R. Schaeffer, A. F. P. Lucena, Air-conditioning and the adaptation cooling deficit in emerging economies. *Nat. Commun.* **12**, 6460 (2021).

71. K. Lundgren, T. Kjellstrom, Sustainability challenges from climate change and air conditioning use in urban areas. *Sustainability* **5**, 3116–3128 (2013).

72. Z. Sultana, H. L. P. Mees, B. Mallick, P. P. J. Driessen, A. Bailey, Rising temperatures, rising vulnerabilities: Individual and community adaptation to heat stress in urban slums of Bangladesh. *Sustain. Cities Soc.* **132**, 106803 (2025).

73. W. M. Thiaw, E. Bekele, S. N. Diouf, D. G. Dewitt, O. Ndiaye, M. K. Ngom Ndiaye, P. N. Ndiaye, N. Diene, M. Diouf, A. Diaw, S. Diop, F. Badj, A. Diouf, Toward experimental heat–health early warning in Africa. *Bull. Am. Meteorol. Soc.* **103**, E1843–E1860 (2022).

74. I. Pinto, R. C. Odoulami, K. A. Lawal, E. Olaniyan, W. A. Ibrahim, K. Guigma, M. Vahlberg, D. Heinrich, C. P. Marghidan, M. Vogel, J. Arrighi, C. Barnes, F. Otto, S. Philip, M. Mistry, S. Sengupta, S. Kew, J. Kimutai, "Dangerous humid heat in southern West Africa about 4°C hotter due to climate change" (Worldwide Weather Attribution, 2024); https://doi.org/10.25561/110082.

75. Red Cross Red Crescent Climate Center, "Urban heat governance: Assessing institutional capacity to manage extreme heat in cities of the Global South" (2026); https://preparecenter.org/resource/urban-heat-governance-assessing-institutional-capacity-to-manage-extreme-heat-in-cities-of-the-global-south/.

76. K. E. Zangerl, K. Hoernke, M. Andreas, S. L. Dalglish, I. Kelman, M. Nilsson, J. Rockloev, T. Bärnighausen, S. A. McMahon, Child health prioritisation in national adaptation policies on climate change: A policy document analysis across 160 countries. *Lancet Child Adolesc. Health* **8**, 532–544 (2024).

77. J. Ubalde, C. Bradshaw, S. H. Fatima, P. Le Souëf, M. Judge, Systematic review of climate change interventions for improving child health. *BMJ Paediatr. Open* **10**, e003415 (2026).

78. UNICEF, *A Threat to Progress: Confronting the Effects of Climate Change on Child Health and Well-Being* (United Nations, Erscheinungsort nicht ermittelbar, 2024).

79. P. U. Bassey, F. A. Ngwu, O. C. Shimobi, A. B. Isiaka, C. C. God'swill, C. Hosein, B. C. Ephraim-Emmanuel, Heat-related illnesses: A scoping review of health system strategies, emergency responses and interventions in heat-prone areas. *BMC Public Health* **25**, 2679 (2025).

80. G. J. Van Oldenborgh, M. F. Wehner, R. Vautard, F. E. L. Otto, S. I. Seneviratne, P. A. Stott, G. C. Hegerl, S. Y. Philip, S. F. Kew, Attributing and projecting heatwaves is hard: We can do better. *Earth's Future* **10**, e2021EF002271 (2022).

81. H. Hersbach, B. Bell, P. Berrisford, S. Hirahara, A. Horányi, J. Muñoz-Sabater, J. Nicolas, C. Peubey, R. Radu, D. Schepers, A. Simmons, C. Soci, S. Abdalla, X. Abellan, G. Balsamo, P. Bechtold, G. Biavati, J. Bidlot, M. Bonavita, G. De Chiara, P. Dahlgren, D. Dee, M. Diamantakis, R. Dragani, J. Flemming, R. Forbes, M. Fuentes, A. Geer, L. Haimberger, S. Healy, R. J. Hogan, E. Hólm, M. Janisková, S. Keeley, P. Laloyaux, P. Lopez, C. Lupu, G. Radnoti, P. De Rosnay, I. Rozum, F. Vamborg, S. Villaume, J. Thépaut, The ERA5 global reanalysis. *Q. J. R. Meteorol. Soc.* **146**, 1999–2049 (2020).

82. C. Brimicombe, F. Conway, A. Portela, D. Lakhoo, N. Roos, C. Gao, I. Solarin, D. Jackson, A scoping review on heat indices used to measure the effects of heat on maternal and perinatal health. *BMJ Public Health* **2**, e000308 (2024).

83. S. Dasgupta, N. Van Maanen, S. N. Gosling, F. Piontek, C. Otto, C.-F. Schleussner, Effects of climate change on combined labour productivity and supply: An empirical, multi-model study. *Lancet Planet. Heath* **5**, e455–e465 (2021).

84. J. K. Vanos, J. W. Baldwin, O. Jay, K. L. Ebi, Simplicity lacks robustness when projecting heat-health outcomes in a changing climate. *Nat. Commun.* **11**, 6079 (2020).

85. G. M. Budd, Wet-bulb globe temperature (WBGT)—Its history and its limitations. *J. Sci. Med. Sport* **11**, 20–32 (2008).

86. L. G. Ioannou, L. Tsoutsoubi, K. Mantzios, M. Vliora, E. Nintou, J. F. Piil, S. R. Notley, P. C. Dinas, G. A. Gourzoulidis, G. Havenith, M. Brearley, I. B. Mekjavic, G. P. Kenny, L. Nybo, A. D. Flouris, Indicators to assess physiological heat strain—Part 3: Multi-country field evaluation and consensus recommendations. *Temperature* **9**, 274–291 (2022).

87. C. Brimicombe, K. Wieser, T. Monthaler, D. Jackson, J. De Bont, M. F. Chersich, I. M. Otto, Effects of ambient heat exposure on risk of all-cause mortality in children younger than 5 years in Africa: A pooled time-series analysis. *Lancet Planet. Heath* **8**, e640–e646 (2024).

88. C. Brimicombe, C. Gao, I. M. Otto, Vulnerable to heat stress: Gaps in international standard metric thresholds. *Int. J. Biometeorol.* **68**, 2495–2506 (2024).

89. Q. Kong, M. Huber, Explicit calculations of wet-bulb globe temperature compared with approximations and why it matters for labor productivity. *Earth's Future* **10**, e2021EF002334 (2022).

90. J. P. Dunne, R. J. Stouffer, J. G. John, Reductions in labour capacity from heat stress under climate warming. *Nat. Clim. Change* **3**, 563–566 (2013).

91. J. C. Liljegren, R. A. Carhart, P. Lawday, S. Tschopp, R. Sharp, Modeling the wet bulb globe temperature using standard meteorological measurements. *J. Occup. Environ. Hyg.* **5**, 645–655 (2008).

92. B. Lemke, T. Kjellstrom, Calculating workplace WBGT from meteorological data: A tool for climate change assessment. *Ind. Health* **50**, 267–278 (2012).

93. Q. Kong, M. Huber, A new, zero-iteration analytic implementation of wet-bulb globe temperature: development, validation and comparison with other methods. *GeoHealth* **8**, e2024GH001068 (2024).

94. C. Schwingshackl, J. Sillmann, A. M. Vicedo-Cabrera, M. Sandstad, K. Aunan, Heat stress indicators in CMIP6: Estimating future trends and exceedances of impact-relevant thresholds. *Earth's Future* **9**, e2020EF001885 (2021).

95. C. H. Simpson, O. Brousse, K. L. Ebi, C. Heaviside, Commonly used indices disagree about the effect of moisture on heat stress. *npj Clim. Atmos. Sci.* **6**, 78 (2023).

96. C. Li, Y. Sun, F. Zwiers, D. Wang, X. Zhang, G. Chen, H. Wu, Rapid warming in summer wet bulb globe temperature in China with human-induced climate change. *J. Climate* **33**, 5697–5711 (2020).

97. C. Li, X. Zhang, F. Zwiers, Y. Fang, A. M. Michalak, Recent very hot summers in northern hemispheric land areas measured by wet bulb globe temperature will be the norm within 20 years. *Earth's Future* **5**, 1203–1216 (2017).

98. D. Li, J. Yuan, R. E. Kopp, Escalating global exposure to compound heat-humidity extremes with warming. *Environ. Res. Lett.* **15**, 064003 (2020).

99. Y. Epstein, D. S. Moran, Thermal comfort and the heat stress indices. *Ind. Health* **44**, 388–398 (2006).

100. C. D. W. Rogers, R. A. Warren, Fast and accurate calculation of wet-bulb temperature for humid-heat extremes. ESS Open Archive [Preprint] (2024); https://doi.org/10.22541/essoar.170560423.39769387/v1.

101. K. Parsons, Heat stress standard ISO 7243 and its global application. *Ind. Health* **44**, 368–379 (2006).

102. J. R. Buzan, Implementation and evaluation of wet bulb globe temperature within non-urban environments in the community land model version 5. *J. Adv. Model Earth Syst.* **16**, e2023MS003704 (2024).

103. K. Frieler, J. Volkholz, S. Lange, J. Schewe, M. Mengel, M. Del Rocío Rivas Lopez, C. Otto, C. P. O. Reyer, D. N. Karger, J. T. Malle, S. Treu, C. Menz, J. L. Blanchard, C. S. Harrison, C. M. Petrik, T. D. Eddy, K. Ortega-Cisneros, C. Novaglio, Y. Rousseau, R. A. Watson, C. Stock, X. Liu, R. Heneghan, D. Tittensor, O. Maury, M. Büchner, T. Vogt, T. Wang, F. Sun, I. J. Sauer, J. Koch, I. Vanderkelen, J. Jägermeyr, C. Müller, S. Rabin, J. Klar, I. D. Vega Del Valle, G. Lasslop, S. Chadburn, E. Burke, A. Gallego-Sala, N. Smith, J.

Chang, S. Hantson, C. Burton, A. Gädeke, F. Li, S. N. Gosling, H. M. Schmied, F. Hattermann, J. Wang, F. Yao, T. Hickler, R. Marcé, D. Pierson, W. Thiery, D. Mercado-Bettín, R. Ladwig, A. I. Ayala-Zamora, M. Forrest, M. Bechtold, Scenario setup and forcing data for impact model evaluation and impact attribution within the third round of the inter-sectoral impact model intercomparison project (ISIMIP3a). *Geosci. Model Dev.* **17**, 1–51 (2024).

104. S. Lange, M. Mengel, S. Treu, M. Büchner, ISIMIP3a atmospheric climate input data, version 1.2, ISIMIP Repository (2022); https://doi.org/10.48364/ISIMIP.982724.2.

105. K. Frieler, S. Lange, J. Schewe, M. Mengel, S. Treu, C. Otto, J. Volkholz, C. P. O. Reyer, S. Heinicke, C. Jones, J. L. Blanchard, C. S. Harrison, C. M. Petrik, T. D. Eddy, K. Ortega-Cisneros, C. Novaglio, R. Heneghan, D. P. Tittensor, O. Maury, M. Büchner, T. Vogt, D. Quesada Chacón, K. Emanuel, C.-Y. Lee, S. J. Camargo, J. Jägermeyr, S. Rabin, J. Klar, I. D. Vega Del Valle, L. Novak, I. J. Sauer, G. Lasslop, S. Chadburn, E. Burke, A. Gallego-Sala, N. Smith, J. Chang, S. Hantson, C. Burton, A. Gädeke, F. Li, S. N. Gosling, H. Müller Schmied, F. Hattermann, T. Hickler, R. Marcé, D. Pierson, W. Thiery, D. Mercado-Bettín, R. Ladwig, A. I. Ayala-Zamora, M. Forrest, M. Bechtold, R. Reinecke, I. De Graaf, J. O. Kaplan, A. Koch, M. Lengaigne, Scenario set-up and the new CMIP6-based climate-related forcings provided within the third round of the Inter-Sectoral Model Intercomparison Project (ISIMIP3b, group I and II). EGUsphere [Preprint] (2025); https://doi.org/10.5194/egusphere-2025-2103.

106. S. Lange, M. Büchner, ISIMIP3b bias-adjusted atmospheric climate input data, version 1.1, ISIMIP Repository (2021); https://doi.org/10.48364/ISIMIP.842396.1.

107. S. Lange, "ISIMIP3b bias adjustment fact sheet" (ISIMIP, 2021); www.isimip.org/documents/413/ISIMIP3b_bias_adjustment_fact_sheet_Gnsz7CO.pdf.

108. S. Philip, S. Kew, G. J. van Oldenborgh, F. Otto, R. Vautard, K. van der Wiel, A. King, F. Lott, J. Arrighi, R. Singh, M. van Aalst, A protocol for probabilistic extreme event attribution analyses. *Adv. Stat. Climatol. Meteorol. Oceanogr.* **6**, 177–203 (2020).

109. P. M. Forster, C. Smith, T. Walsh, W. F. Lamb, R. Lamboll, B. Hall, M. Hauser, A. Ribes, D. Rosen, N. P. Gillett, M. D. Palmer, J. Rogelj, K. Von Schuckmann, B. Trewin, M. Allen, R. Andrew, R. A. Betts, A. Borger, T. Boyer, J. A. Broersma, C. Buontempo, S. Burgess, C. Cagnazzo, L. Cheng, P. Friedlingstein, A. Gettelman, J. Gütschow, M. Ishii, S. Jenkins, X. Lan, C. Morice, J. Mühle, C. Kadow, J. Kennedy, R. E. Killick, P. B. Krummel, J. C. Minx, G. Myhre, V. Naik, G. P. Peters, A. Pirani, J. Pongratz, C.-F. Schleussner, S. I. Seneviratne, S. Szopa, P. Thorne, M. V. M. Kovilakam, E. Majamäki, J.-P. Jalkanen, M. Van Marle, R. M. Hoesly, R. Rohde, D. Schumacher, G. Van Der Werf, R. Vose, K. Zickfeld, X. Zhang, V. Masson-Delmotte, P. Zhai, Indicators of Global Climate Change 2023: Annual update of key indicators of the state of the climate system and human influence. *Earth Syst. Sci. Data* **16**, 2625–2658 (2024).

110. C. Tebaldi, J. M. Arblaster, Pattern scaling: Its strengths and limitations, and an update on the latest model simulations. *Clim. Change* **122**, 459–471 (2014).

111. N. Herger, B. M. Sanderson, R. Knutti, Improved pattern scaling approaches for the use in climate impact studies. *Geophys. Res. Lett.* **42**, 3486–3494 (2015).

112. M. Hauser, L. Gudmundsson, R. Orth, A. Jézéquel, K. Haustein, R. Vautard, G. J. Van Oldenborgh, L. Wilcox, S. I. Seneviratne, Methods and model dependency of extreme event attribution: The 2015 European drought. *Earth's Future* **5**, 1034–1043 (2017).

113. M. Hauser, mathause/dist_cov: version 0.1.0, version v0.1.0, Zenodo (2023); https://doi.org/10.5281/ZENODO.7922001.

114. K. Haustein, M. R. Allen, P. M. Forster, F. E. L. Otto, D. M. Mitchell, H. D. Matthews, D. J. Frame, A real-time global warming index. *Sci. Rep.* **7**, 15417 (2017).

115. F. E. L. Otto, D. J. Frame, A. Otto, M. R. Allen, Embracing uncertainty in climate change policy. *Nat. Clim. Change* **5**, 917–920 (2015).

116. S. Lange, Trend-preserving bias adjustment and statistical downscaling with ISIMIP3BASD (v1.0). *Geosci. Model Dev.* **12**, 3055–3070 (2019).

117. M. Mengel, S. Treu, S. Lange, K. Frieler, ATTRICI v1.1—Counterfactual climate for impact attribution. *Geosci. Model Dev.* **14**, 5269–5284 (2021).

118. Wittgenstein Center, Wittgenstein Centre Human Capital Data Explorer v2, version 2; https://dataexplorer.wittgensteincentre.org/wcde-v2/.

119. J. Volkholz, S. Lange, I. Sauer, C. Otto, ISIMIP3a population input data, version 1.3, ISIMIP Repository (2024); https://doi.org/10.48364/ISIMIP.822480.3.

120. J. Volkholz, S. Lange, I. Sauer, C. Otto, ISIMIP3b population input data, version 1.2, ISIMIP Repository (2024); https://doi.org/10.48364/ISIMIP.889136.2.

121. K. Klein Goldewijk, A. Beusen, J. Doelman, E. Stehfest, Anthropogenic land use estimates for the Holocene–HYDE 3.2. *Earth Syst. Sci. Data* **9**, 927–953 (2017).

122. Department of Economic and Social Affairs: Population Division, *World Population Prospects 2019* (United Nations, 2019); www.un.org/development/desa/pd/news/world-population-prospects-2019-0.

123. J. Gao, Global 1-km Downscaled Population Base Year and Projection Grids for the Shared Socioeconomic Pathways (SSPs), Revision 01, version 1.01, Palisades, NY: NASA Socioeconomic Data and Applications Center (SEDAC) (2020); https://doi.org/10.7927/Q7Z9-9R69.

124. B. Jones, B. C. O'Neill, Spatially explicit global population scenarios consistent with the Shared Socioeconomic Pathways. *Environ. Res. Lett.* **11**, 084003 (2016).

125. M. Bondarenko, R. Priyatikanto, N. Tejedor-Garavito, W. Zhang, T. McKeen, A. Cunningham, T. Woods, J. Hilton, D. Cihan, B. Nosatiuk, T. Brinkhoff, A. Tatem, A. Sorichetta, The spatial distribution of population broken down by gender and age groupings in 2015-2030 at a resolution of 30 arc (approximately 1km at the equator). Global Demographic Data Project - Funded by The Bill and Melinda Gates Foundation (INV-045237)., version R2025A version v1, WorldPop - School of Geography and Environmental

Science, University of Southampton (2025); https://doi.org/DOI:10.5258/SOTON/WP00846.

126. K. Riahi, D. P. Van Vuuren, E. Kriegler, J. Edmonds, B. C. O'Neill, S. Fujimori, N. Bauer, K. Calvin, R. Dellink, O. Fricko, W. Lutz, A. Popp, J. C. Cuaresma, S. Kc, M. Leimbach, L. Jiang, T. Kram, S. Rao, J. Emmerling, K. Ebi, T. Hasegawa, P. Havlik, F. Humpenöder, L. A. Da Silva, S. Smith, E. Stehfest, V. Bosetti, J. Eom, D. Gernaat, T. Masui, J. Rogelj, J. Strefler, L. Drouet, V. Krey, G. Luderer, M. Harmsen, K. Takahashi, L. Baumstark, J. C. Doelman, M. Kainuma, Z. Klimont, G. Marangoni, H. Lotze-Campen, M. Obersteiner, A. Tabeau, M. Tavoni, The shared socioeconomic pathways and their energy, land use, and greenhouse gas emissions implications: An overview. *Glob. Environ. Chang.* **42**, 153–168 (2017).

127. C. J. Smith, P. M. Forster, M. Allen, N. Leach, R. J. Millar, G. A. Passerello, L. A. Regayre, FAIR v1.3: A simple emissions-based impulse response and carbon cycle model. *Geosci. Model Dev.* **11**, 2273–2297 (2018).

128. O. Fricko, P. Havlik, J. Rogelj, Z. Klimont, M. Gusti, N. Johnson, P. Kolp, M. Strubegger, H. Valin, M. Amann, T. Ermolieva, N. Forsell, M. Herrero, C. Heyes, G. Kindermann, V. Krey, D. L. McCollum, M. Obersteiner, S. Pachauri, S. Rao, E. Schmid, W. Schoepp, K. Riahi, The marker quantification of the Shared Socioeconomic Pathway 2: A middle-of-the-road scenario for the 21st century. *Glob. Environ. Chang.* **42**, 251–267 (2017).

129. F. E. L. Otto, C. Barnes, S. Philip, S. Kew, G. J. Van Oldenborgh, R. Vautard, Formally combining different lines of evidence in extreme-event attribution. *Adv. Stat. Clim. Meteorol. Oceanogr*. **10**, 159–171 (2024).
